# Identification of the Polymerizing Glycosyltransferase Required for the Addition of d-Glucuronic Acid to the Capsular Polysaccharide of *Campylobacter jejuni*

**DOI:** 10.1021/acs.biochem.4c00703

**Published:** 2025-01-24

**Authors:** Dao Feng Xiang, Alexander S. Riegert, Tamari Narindoshvili, Frank M. Raushel

**Affiliations:** † Department of Biochemistry & Biophysics, Texas A&M University, College Station, Texas 77843, United States; ‡ Department of Chemistry, Texas A&M University, College Station, Texas 77843, United States

## Abstract

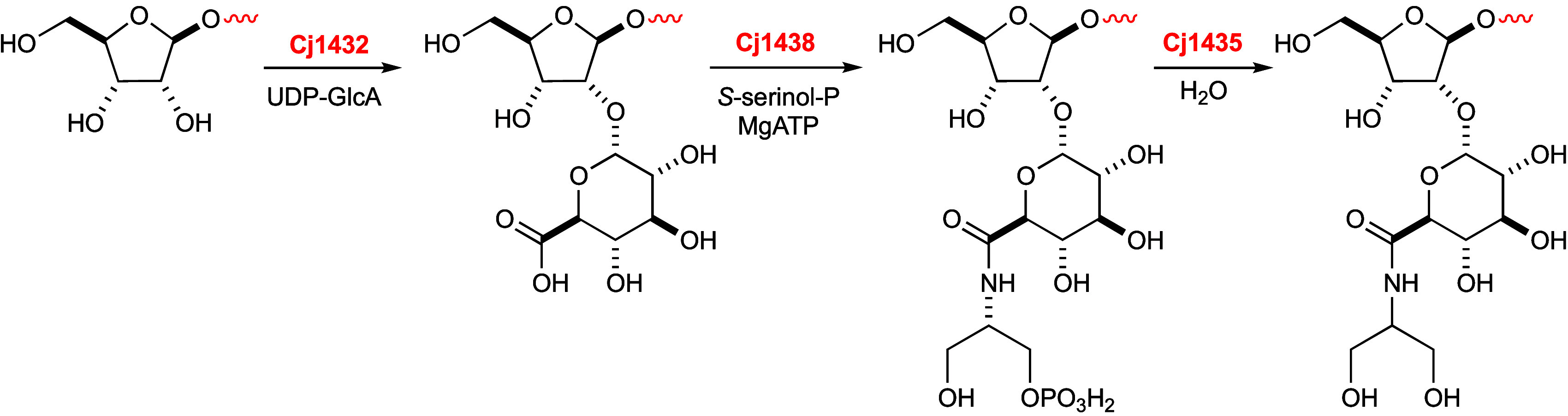

*Campylobacter jejuni* is the leading cause of food poisoning in Europe and North America. The exterior surface of this bacterium is encased by a capsular polysaccharide that is attached to a diacyl glycerol phosphate anchor via a poly-Kdo (3-deoxy-d-*manno*-oct-2-ulosinic acid) linker. In the HS:2 serotype of *C. jejuni* NCTC 11168, the repeating trisaccharide consists of d-ribose, *N*-acetyl-d-glucosamine, and d-glucuronate. Here, we show that the N-terminal domain of Cj1432 (residues 1–356) is responsible for the reaction of the C2 hydroxyl group from the terminal d-ribose moiety of the growing polysaccharide chain with UDP-d-glucuronate as the donor substrate. This discovery represents the first biochemical identification and functional characterization of a glycosyltransferase responsible for the polymerization of the capsular polysaccharide of *C. jejuni*. The product of the reaction catalyzed by the N-terminal domain of Cj1432 is the substrate for the reaction catalyzed by the C-terminal domain of Cj1438 (residues 453–776). This enzyme catalyzes amide bond formation using the C6 carboxylate of the terminal d-glucuronate moiety and (*S*)-serinol phosphate as substrates. It is also shown that Cj1435 catalyzes the hydrolysis of phosphate from the product catalyzed by the C-terminal domain of Cj1438. These results demonstrate that amide decoration of the d-glucuronate moiety occurs after the incorporation of this sugar into the growing polysaccharide chain.

## Introduction

*Campylobacter jejuni* is a leading foodborne pathogen that causes gastroenteritis in humans. This infection accounts for >400 million cases of diarrhea each year worldwide.^1^ The main routes of transmission are generally believed to be associated with undercooked poultry, raw milk, and contaminated water. Individuals with *Campylobacter* infections usually have diarrhea, fever, vomiting, and stomach cramps.^2^ Additionally, campylobacteriosis can result in a rare autoimmune disease known as Guillain-Barré Syndrome (GBS).^3^ It is estimated that 40% of all new GBS cases are preceded by a *Campylobacter* infection.^4^ Currently, there are no FDA-approved vaccines for the prevention of *Campylobacte*r infection. So far, the best candidates are conjugate vaccines, which mimic the surface-exposed capsular polysaccharides (CPS).^5^

The various strains and serotypes of *C. jejuni* synthesize structurally different capsular polysaccharides on the exterior cell surface that help to protect them from the host immune response.^6^ The CPS is also important for structural stability and maintenance of the bacterial cell wall.^7^ Deletion of the gene clusters required for the biosynthesis of the CPS diminishes the pathogenicity of *C. jejuni*, and thus, the enzymes responsible for the biosynthesis of these essential polysaccharides are potential therapeutic targets.^7^ The capsular polysaccharides from *C. jejuni* are composed of a repeating series of monosaccharide units attached to one another via glycosidic bonds. The carbohydrates can be further decorated with a variety of modifications including methylation, amidation, and the addition of O-methyl phosphoramidate (MeOPN).^6,8^ The chemical compositions of at least 12 unique CPS structures from more than 33 different *C. jejuni* serotypes have been identified thus far.^6,9^

**Figure 1:**
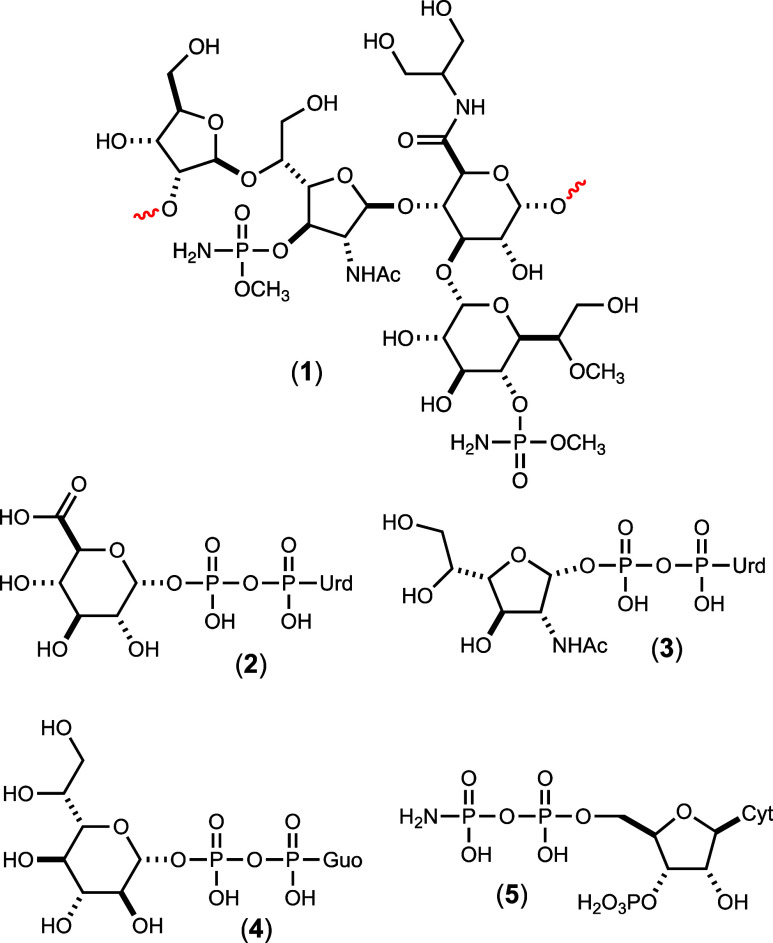
Structure of the repeating polysaccharide (**1**) identified in the CPS of *C. jejuni* NCTC 11168 (serotype HS:2). The lipid anchor is attached to the reducing end (right side) of the structure, as drawn. Also shown are the three nucleotide sugars (UDP-GlcA; UDP-GalfNAc; and GDP-d-*glycero*-l-*gluco*-heptose) previously identified as the carbohydrate donors for the assembly of the capsular polysaccharide.^11−13^ Also shown is the likely donor for the phosphoramidate modification (**5**).^14^.

The structure of the repeating polysaccharide (**1**) unit in the CPS of the most studied strain of *C. jejuni* (NCTC 11168; serotype HS:2) is presented in Figure 1 in addition to three nucleotide sugars for glycosyltransfer to the growing CPS. The CPS consists of a linear trisaccharide of d-ribose (d-Rib), *N*-acetyl-d-galactosamine (d-Gal*f*NAc), and d-glucuronic acid (d-GlcA) with a branching unit of d-*glycero*-l-*gluco*-heptose that is attached to a lipid anchor at the reducing end of the polysaccharide.^6^ The d-*glycero*-l-*gluco*-heptose moiety is further decorated by substitution with a methyl phosphoramidate (MeOPN) modification at C4 and methylation at C6.^6,10^ A MeOPN decoration is also found at C3 of the d-Gal*f*NAc moiety. The d-glucuronic acid is further amidated with either serinol (as shown) or ethanolamine (not shown).

**Figure 2:**
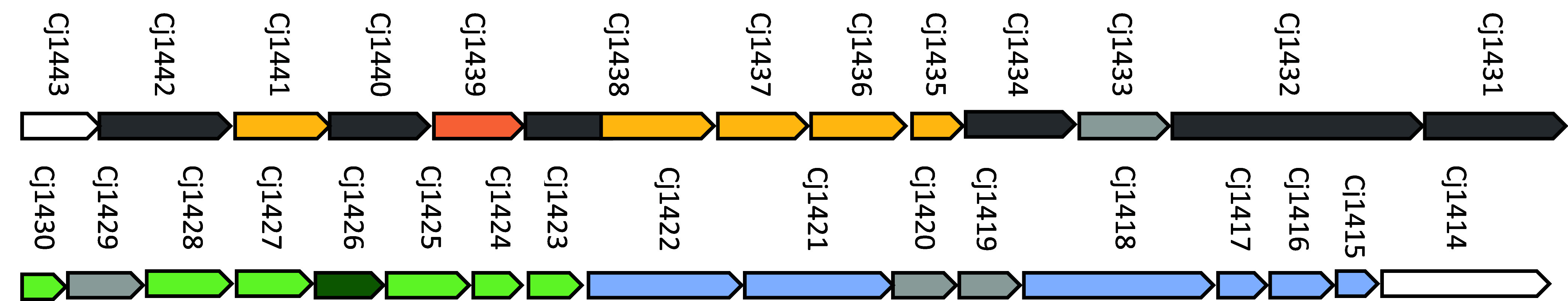
Gene cluster for the biosynthesis of the CPS in *C. jejuni* NCTC 11168 (serotype HS:2).^27^ Light gray: no current functional assignment; light blue: synthesis of the methyl phosphoramidate; light green: synthesis of GDP-d-*glycero*-l-*gluco*-heptose; dark green: methylation of d-*glycero*-l-*gluco*-heptose; light yellow: synthesis of UDP-GlcA and subsequent amide bond formation; pink: UDP-Gal*p*NAc mutase; dark gray: glycosyltransferases.

A portion of the gene cluster that contains most (but not all) of the genes required for the biosynthesis of the CPS from *C. jejuni* NCTC 11168 is presented in Figure 2. Many of the reactions catalyzed by the enzymes encoded by the genes from this cluster have been experimentally ascertained by *in vitro* characterization of the purified proteins or via analysis of the resultant capsular polysaccharide after deletion of specific genes.^11−26^ No functional assignments have been made for Cj1419, Cj1420, Cj1429, or Cj1433 since gene knockouts resulted in no apparent changes to the CPS structure.^18^ However, deletions of Cj1421 and Cj1422 resulted in the loss of the MeOPN modification to the d-GalNAc and heptose moieties, respectively, and mutation of the gene for Cj1426 negated the methylation of the d-*glycero*-l-*gluco*-heptose moiety.^19^ Knockout mutations of most of the remaining genes (except for Cj1436, Cj1415, Cj1416, Cj1417, and Cj1418) resulted in an acapsular phenotype.

*In vitro* characterization of purified enzymes has established functional significance for many of the remaining enzymes. Cj1441 was shown to catalyze the NAD^+^-dependent oxidation of UDP-d-Glc to UDP-d-GlcA (**2**) and Cj1439 was required for the conversion of UDP-d-Gal*p*NAc to UDP-d-Gal*f*NAc (**3**).^11,12^ Collectively, Cj1423, Cj1424, Cj1425, Cj1427, Cj1428, and Cj1430 were shown to be required to make GDP-d-*glycero*-l-*gluco*-heptose (**4**) from d-sedoheptulose-7-phosphate, and Cj1415, Cj1416, Cj1417, and Cj1418 were required to make CDP-phosphoramidate 3′-phosphate (**5**) from l-glutamine, ATP, and CTP.^13,14,20−23,28−34^ The remaining functionally characterized enzymes include Cj1437 (transamination of dihydroxyacetone phosphate to (*S*)-serinol-P) and Cj1436 (decarboxylation of l-serine-phosphate to ethanolamine-P).^24,25^ The C-terminal domain of Cj1438 (residues 543–776) was shown to catalyze the ATP-dependent amidation of the methyl glycoside of d-glucuronic acid with either (*S*)-serinol-P or ethanolamine-P, and Cj1435 was shown to catalyze the hydrolysis of phosphate from the resulting product.^25,26^

The biosynthesis of the CPS within *C. jejuni* is currently thought to follow the ABC transporter-dependent pathway.^35^ In this pathway, the enzyme KpsS uses CMP-Kdo to add a single Kdo to a diacylglycerophosphate acceptor while the bifunctional enzyme KpsC adds multiple Kdo units to this product within the cytosol.^35^ The KpsC-catalyzed product is the initial acceptor substrate for the array of glycosyltransferases that are needed to synthesize the capsular polysaccharides via the subsequent addition of alternating sugar moieties. The diacylglycerophosphate anchor is then transported to the outer membrane. The distinctive carbohydrate sequence of the CPS is governed by the substrate specificities of the expressed glycosyltransferases.

The remaining functionally significant, but uncharacterized, enzymes in the HS:2 serotype of *C. jejuni* include Cj1442, Cj1440, Cj1438 (N-terminal half), Cj1434, and Cj1432. Included within this list are the three glycosyltransferases that are the required polymerases that must add d-glucuronic acid, d-ribose, and d-Gal*f*NAc to the growing polysaccharide chain, and the glycosyltransferase that initiates polymerization by adding a specific monosaccharide to the poly-Kdo linker.^36−39^ Here, we functionally characterized the first glycosyltransferase required for the polymerization of the growing polysaccharide chain from the CPS of *C. jejuni*. The N-terminal domain of Cj1432 is shown to catalyze the transfer of d-glucuronic acid from UDP-GlcA (**2**) to C2 of a d-ribofuranoside acceptor with retention of the configuration at the anomeric carbon.

## Materials and Methods

### Materials

UDP-d-GlcA (**2**) was purchased from Carbosynth and methyl 2-deoxy-d-riboside was obtained from Sigma-Aldrich. Methyl β-d-riboside (**6a**), d-[2-^13^C]-ribose, and d-[3-^13^C]-ribose were obtained from Omicron. Lysogeny broth (LB) and isopropyl-β-d-thiogalactopyranoside (IPTG) were purchased from Research Products International. HisTrap columns, HiTrap Q HP anion exchange columns, and Vivaspin 20 10 kDa MWCO spin filters were obtained from Cytiva. The 10K Nanosep spin filters were purchased from PALL Corporation (Port Washington, NY). The protease inhibitor cocktail (cOmplete Mini), DNase I, kanamycin, ampicillin, imidazole, D_2_O (99.9 atom %), and HEPES were purchased from Sigma-Aldrich. All other compounds, unless stated otherwise, were purchased from either Sigma-Aldrich or Thermo Fisher Scientific.

### Equipment

Nuclear magnetic resonance (NMR) spectra were recorded at room temperature using standard pulse sequences on either a Bruker Avance III 400 MHz NMR spectrometer or an Avance III 500 MHz NMR spectrometer equipped with a broad band probe and sample changer. Electrospray ionization mass spectrometry (ESI-MS) experiments were performed using a Thermo Scientific Q Exactive Focus. Samples were injected into a 10 μL loop and transferred to the instrument by using a mobile phase containing 70% methanol and 30% water with 0.1% formic acid at a flow rate of 600 μL/min. The Q Exactive Focus HESI source was operated in full MS in positive and negative modes. The mass resolution was tuned to 70,000 fwhm (full width at half-maximum) at *m*/*z* 200. The spray voltage was set to 3.5 kV for positive mode and 2.8 kV for negative mode. The vaporizer and transfer capillary temperatures were held at 250 and 320 °C, respectively. The S-Lens RF level was set at 50 v. Exactive Series 2.11/Xcalibur 4.2.47 software was used for data acquisition and processing.

### Synthesis of ^13^C-Labeled Methyl β-d-Riboside (**6b, 6c**)

**Figure 3:**
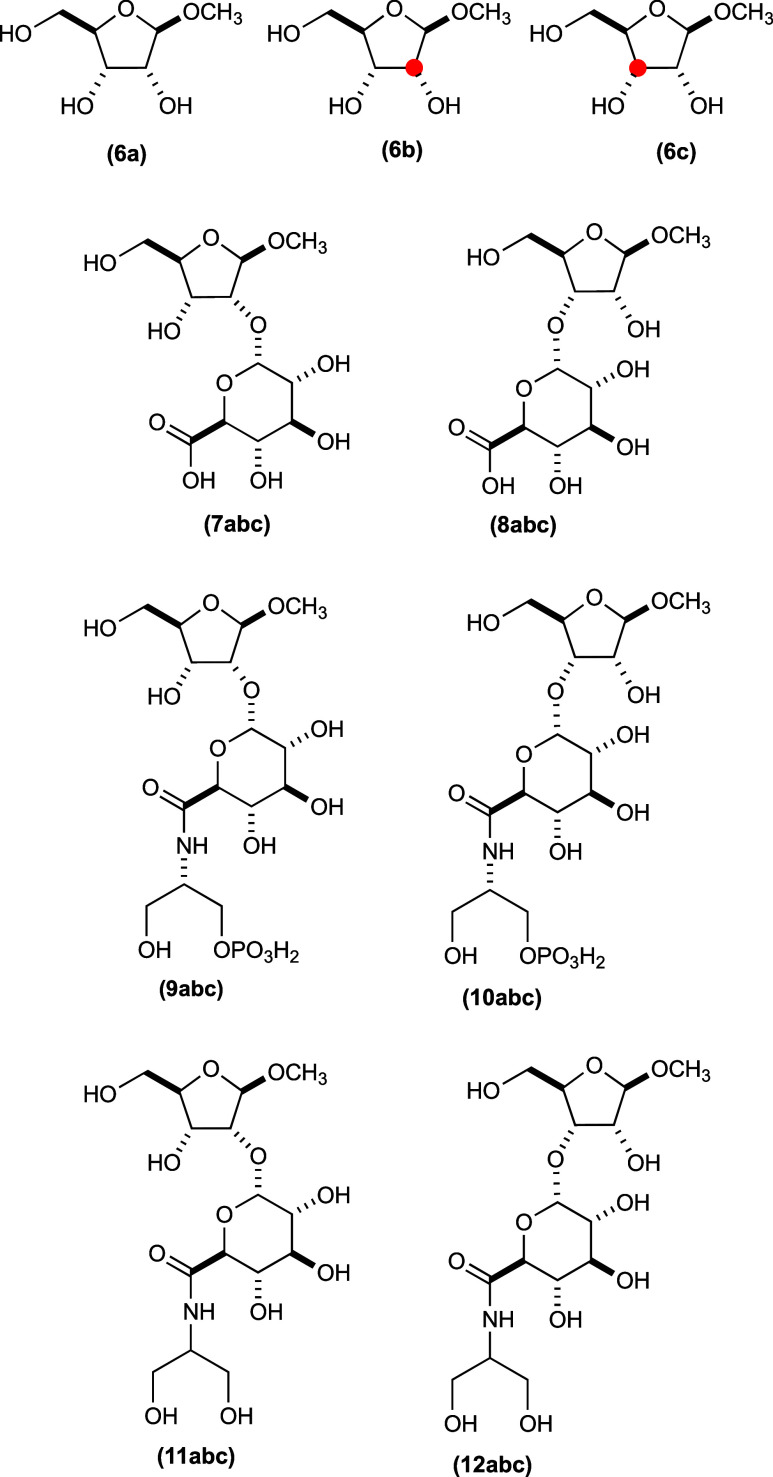
Structures of the substrates and reaction products. Those compounds designated as “**a**” do not have a ^13^C label at either C2 or C3 of the d-ribose moiety. Those compounds designated as “**b**” have a ^13^C label at C2 of the d-ribose moiety, and those designated as “**c**” have a ^13^C label at C3 of the d-ribose moiety.

To a solution of [2-^13^C]- or [3-^13^C]-d-ribose (150 mg, 1.0 mmol) in MeOH (3.0 mL) was added 30 μL of conc. H_2_SO_4_ at 0 °C. The mixture was stirred at 4 °C overnight. The solution was neutralized by the addition of an anion exchange resin (Dowex-OH form) and concentrated to dryness. The product (β-anomer, 80%; α-anomer, 20%) was obtained as a colorless oil in a quantitative yield. The ^1^H NMR spectra of the unlabeled and ^13^C-labeled derivatives of methyl β-d-riboside (**6a**, **6b**, and **6c**) are presented in Figure S1. The structures of the substrates and products made for this investigation are shown in Figure 3.

### Cloning, Expression, and Purification of Cj1432_N_

The gene encoding the N-terminal domain of Cj1432 (residues 1–356; UniProt ID: Q0P8I2) from *C. jejuni* NCTC 11168 was purchased from Twist Biosciences (sequenced by Eton Bioscience) in a pET28a expression vector with an N-terminal hexahistidine tag. The enzyme is denoted as Cj1432_N_ for this investigation. BL21 (DE3) cells (Thermo Fisher Scientific) were transformed with the Cj1432_N_-pET28a plasmid. Single colonies were incubated in 5 mL of LB medium supplemented with 50 μg/mL kanamycin at 37 °C overnight. Each 5 mL starter culture was added to 1 L of LB medium supplemented with 50 μg/mL of kanamycin. Routinely, 6 L cultures of LB medium were grown for purification. Protein expression was induced by the addition of 1.0 mM IPTG when the cell density reached an OD_600_ of 0.6–0.8. The cells were allowed to express at 21 °C for 18 h before they were harvested via centrifugation at 7000 rcf at 4 °C, frozen in liquid N_2_, and stored at −80 °C. In a typical purification of Cj1432_N_, ∼10 g of frozen cell paste was resuspended in 100 mL of 50 mM HEPES, 300 mM KCl, and 10 mM imidazole (pH 8.0), supplemented with 0.05 mg/mL of the protease inhibitor cocktail and 40 U/mL of DNase I. The resuspended cells were lysed by sonication (QSONICA Sonicator Ultrasonic Processor) in an ice bath using an on/off cycle of 10 s at 40% power for a total of 20 min. The lysate was clarified by centrifugation at 30,000 rcf at 4 °C, passed through a 0.45 μm filter (Whatman), and then loaded onto a 5 mL HisTrap column connected to an F10 NGC Chromatography System (Bio-Rad) previously calibrated with buffer A containing 50 mM HEPES, 300 mM KCl, and 10 mM imidazole, pH 8.0. The protein was eluted from the column using a 0–60% linear gradient of buffer B containing 50 mM HEPES, 250 mM KCl, and 500 mM imidazole, pH 8.0, over a total of 30 column volumes (150 mL) at a flow rate of 4.0 mL/min. The fractions containing the desired protein, as identified by SDS gel electrophoresis, were combined. The imidazole was removed by dialysis using buffer C containing 50 mM HEPES and 250 mM KCl, pH 8.0. The protein was concentrated to ∼10 mg/mL, aliquoted, frozen in liquid N_2_, and stored at −80 °C. The concentration of the enzyme was determined spectrophotometrically using a computationally derived molar absorption coefficient at 280 nm.^40^ The values of ε_280_ (M^–1^ cm^–1^) and molecular weight (Da) used for Cj1432_N_ were 64,700 and 43,888, respectively. About 10 mg of protein was obtained per liter of cell culture.

### Cloning, Expression, and Purification of Cj1438_C_

The gene encoding the C-terminal domain of Cj1438 (residues 453–776; UniProt ID: Q0P8H6) from *C. jejuni* NCTC 11168 was previously cloned into a pET31b(+) expression vector with a C-terminal hexahistidine tag.^26^ This protein is denoted as Cj1438_C_ in this investigation. The conditions and procedures for expression and purification of Cj1438_C_ were the same as that for Cj1432_N_ except that ampicillin was used instead of kanamycin during cell growth. The concentration of the purified Cj1438_C_ protein was determined spectrophotometrically using a computationally derived molar absorption coefficient at 280 nm.^40^ The values of ε_280_ (M^–1^ cm^–1^) and molecular weight (Da) used for Cj1438_C_ were 64,500 and 40,084, respectively. The protein was concentrated to ∼20 mg/mL before being flash-frozen in liquid nitrogen and stored at −80 °C. Approximately 35 mg of protein was obtained per liter of cell culture.

### Cloning, Expression, and Purification of Cj1435

The gene encoding Cj1435 (UniProt ID: Q0P8H9) from *C. jejuni* NCTC 11168 was previously cloned into a pET31b expression vector with a C-terminal hexahistidine tag.^25^ The conditions and procedures for expression and purification of this enzyme were the same as those for Cj1438_C_. The concentration of purified Cj1435 was determined spectrophotometrically using a computationally derived molar absorption coefficient of 280 nm.^40^ The values of ε_280_ (M^–1^ cm^–1^) and molecular weight used for Cj1435 were 34,500 and 25,755, respectively. The protein was concentrated to 6 mg/mL before being flash-frozen in liquid nitrogen and stored at −80 °C. Representative SDS gels of the purified proteins are presented in Figure S2.

### Design of Cj1432 and Cj1438 Truncations

**Figure 4:**
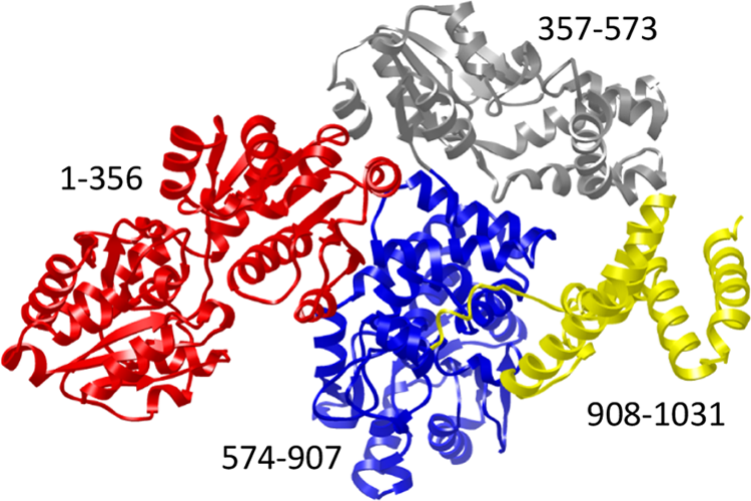
AlphaFold2 generated structure of Cj1432.^41^ The three-dimensional structure of the red domain (residues 1–356) corresponds to a GT4 glycosyltransferase. The domains colored blue and gray are predicted to catalyze the transfer of d-ribose-5-P from PRPP to the growing polysaccharide chain. The yellow domain has an unknown function.

**Figure 5:**
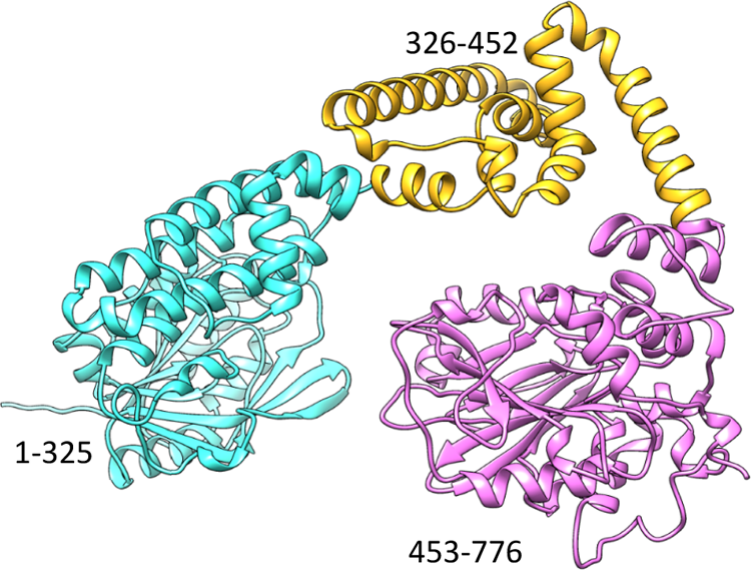
AlphaFold2 generated structure of Cj1438.^41^ The three-dimensional structure of the light blue domain (residues 1–325) corresponds to a GT2 glycosyltransferase. The pink domain (residues 453–776) has been shown previously to catalyze the ATP-dependent amide bond formation between d-glucuronate and (*S*)-serinol-P.^26^ The yellow domain is of an unknown function.

The truncated protein used for this investigation, Cj1432_N_, was initially designed using AlphaFold2 to determine the potential boundaries of functionally distinct domains.^41^ The PDB coordinate file for Cj1432 (AF-Q0P8I2-F1-model_v4.pdb) was downloaded from the AlphaFold database (https://alphafold.ebi.ac.uk). The predicted structures were visualized using USCF Chimera, and the sites of truncation were chosen to maximize the probability of a soluble and catalytically active product. The AlphaFold2 predicted structure of Cj1432 is shown in Figure 4, and the N-terminal domain for Cj1432_N_ is highlighted in red. The expression plasmid for Cj1438_C_ was constructed as described previously and the AlphaFold2 predicted structure of Cj1438 (AF-Q0P8H6-F1-model_v4.pdb) is presented in Figure 5 with the C-terminal domain highlighted in pink. The amino acid sequences for the three proteins purified for this investigation are listed in Figure S3.

### Catalytic Activity of Cj1432_N_

The catalytic activity of Cj1432_N_ was initially tested using UDP-d-GlcA (**2**) as the donor substrate and d-ribose, d-ribose 5-P, methyl β-d-riboside (**6a**), methyl 2-deoxy-β-d-riboside, and methyl β-d-riboside-5-P as potential acceptor substrates. Cj1432_N_ (50 μM) was incubated with 5.0 mM UDP-d-GlcA and 20 mM d-ribose derivatives in 50 mM NH_4_HCO_3_, pH 8. 0 in a volume of 1.0 mL. The reaction mixtures were incubated at 25 °C overnight. The protein was removed using 10K Nanosep spin filters (PALL) prior to mass spectrometric analysis.

### Identification of Reaction Products

The products of the reaction catalyzed by Cj1432_N_ were identified by electrospray ionization mass spectrometry (ESI-MS) and ^1^H NMR spectroscopy. The reaction mixture contained 5.0 mM UDP-d-GlcA (**2**) and 20 mM methyl β-d-riboside (**6a, 6b**, or **6c**) in 50 mM NH_4_HCO_3_ buffer, pH 8.0. The reaction was initiated by the addition of 50 μM Cj1432_N_ and allowed to incubate at 25 °C for 18 h before the enzyme was removed by filtration using a 10K Nanosep spin filter (PALL). The reaction mixture was loaded onto a 5 mL HiTrap Q HP anion exchange column connected to an F10 NGC Chromatography System (Bio-Rad) and washed thoroughly with water (50 mL). The product was eluted from the column using a linear gradient (150 mL) of NH_4_HCO_3_ (0–60% of 500 mM NH_4_HCO_3_, pH 8.0). The individual fractions were analyzed using mass spectrometry (Thermo Scientific Q Exactive Focus mass spectrometer). The fractions containing the product were pooled, lyophilized, and dissolved in D_2_O for ESI-MS and ^1^H NMR analysis.

### Catalytic Activity of Cj1438_C_

The product of the reaction catalyzed by Cj1438_C_ was investigated using ESI-MS and ^1^H NMR spectroscopy. Cj1438_C_ (10 μM) was incubated for 4 h with 5.0 mM ATP, 5.0 mM (*S*)-serinol phosphate, 5.0 mM Cj1432_N_ catalyzed reaction product (either **7a**, **7b**, or **7c**), 10 mM MgCl_2_, and 50 mM NH_4_HCO_3_ buffer (pH 8.0). The reaction was allowed to incubate at 25 °C and monitored using ^31^P NMR spectroscopy. The reaction mixture was passed through a 10K Nanosep spin filter (PALL) to remove the enzyme before being diluted to 15 mL and loaded onto a 5 mL HiTrap Q HP anion exchange column connected to an NGC Chromatography System (Bio-Rad) and washed thoroughly with water (50 mL). The Cj1438_C_ reaction product was eluted from the column using a linear gradient (150 mL) of NH_4_HCO_3_ (0–60% of 500 mM NH_4_HCO_3_). The fractions were analyzed using ESI-MS. The fractions containing the Cj1438_C_ reaction product were pooled, lyophilized, dissolved in D_2_O, and then analyzed by ESI-MS, ^31^P, and ^1^H NMR spectroscopy.

### Catalytic Activity of Cj1435

The Cj1435-catalyzed reaction product was obtained by incubating 5.0 μM Cj1435 and 4.0 mM of the Cj1438_C_ reaction product (**9a**, **9b**, or **9c**) for 4 h in 50 mM NH_4_HCO_3_, pH 8. 0. The reaction was monitored by ^31^P NMR spectroscopy to follow the formation of P_i_.

### Determination of Rate of Reaction Catalyzed by Cj1432_N_

The rate of the Cj1432_N_-catalyzed reaction was determined by monitoring the formation of UDP as a function of time. Cj1432_N_ (10 μM) was incubated with 2.0 mM UDP-d-GlcA (**2**) and 0–25 mM methyl β-d-riboside (**6a**), in 50 mM NH_4_HCO_3_, pH 8.0, at 25 °C. At different time intervals, an aliquot of the reaction mixture was heated to 100 °C for 60 s to denature the protein and quench the reaction. The precipitated protein was removed by centrifugation, and the supernatant solution was passed through a 10K Nanosep spin filter (PALL), diluted in H_2_O, and then loaded onto a 1.0 mL HiTrap Q HP anion exchange column, washed thoroughly with H_2_O, and then eluted from the column with a linear gradient of NH_4_HCO_3_, pH 8.0. The substrate, UDP-d-GlcA (**2**), and UDP product were well separated from one another and quantitated by monitoring the change in absorbance at 255 nm.

## Results and Discussion

### Putative Glycosyltransferases from *C. jejuni* NCTC 11168

Within the gene cluster for the biosynthesis of the CPS from *C. jejuni* NCTC 11168, there are seven putative glycosyltransferase enzymes (Table 1). Prior gene knockout experiments have established that Cj1431 is required for the transfer of d-*glycero*-l-*gluco*-heptose from GDP-d-*glycero*-l-*gluco*-heptose (**4**) to C3 of the d-GlcA moiety of the growing polymeric chain.^18,42^ The six remaining enzymes do not have an assigned function. A GT4 glycosyltransferase can be identified within the N-terminal domain of Cj1432 from residues 1–356 and an AlphaFold2 predicted structure of the entire protein is illustrated in Figure 4, where the N-terminal GT4 domain is highlighted in red.^43,44^ The domains colored gray and blue are likely required for the transfer of d-ribose-5-P from phosphoribosyl pyrophosphate (PRPP) to the d-Gal*f*NAc moiety of the growing polysaccharide chain.^45,46^ This prediction is based on the structural similarity of these two domains to the enzyme that was recently identified for the transfer of d-ribose-5-P from PRPP to the d-ribitol-5-phosphate moiety in the CPS of *Haemophilus influenzae*.^46^ The function of the yellow domain is currently unknown.

**Table 1:** Glycosyltransferase Candidates in the Gene Cluster for CPS Formation in *C. jejuni* NCTC 11168 (HS:2 Serotype)

protein	UniProt ID	length	segment^a^	probable sugar donor, class
Cj1431	Q0P8I3	582	1–471	GDP-d-*glycero*-l-*gluco*-heptose
Cj1432	Q0P8I2	1031	1–356	unknown, GT4
Cj1432	Q0P8I2	1031	357–913	phosphoribosyl pyrophosphate
Cj1434	Q0P8I0	445	1–317	unknown, GT2
Cj1438	Q0P8H6	776	1–325	unknown, GT2
Cj1440	Q0P8H4	407	1–300	unknown, GT2
Cj1442	Q0P8H2	544	1–292	unknown, GT2

a The portion of the multidomain protein that comprises the glycosyltransferase domain as determined by a visual inspection of the calculated three-dimensional structure from AlphaFold2.

Cj1434, Cj1440, and Cj1442 have been classified as GT2 glycosyltransferases by the CAZy database.^47^ The remaining glycosyltransferase, Cj1438, is part of a larger multidomain protein whose AlphaFold2 predicted structure is presented in Figure 5. We have previously shown that the C-terminal domain of Cj1438 (residues 453–776) is responsible for amide bond formation between d-GlcA and (*S*)-serinol-P.^26^ The domain that extends from residues 1–325 is a GT2 glycosyltransferase.

Of the seven glycosyltransferases identified in the gene cluster for CPS formation in *C. jejuni* NCTC 11168, the most likely candidate for the formation of the glycosidic bond between d-GlcA and d-Rib with retention of configuration at C1 is the N-terminal domain of Cj1432. This is the only glycosyltransferase that is annotated as a GT4 glycosyltransferase, and these enzymes are known to catalyze the reaction with retention of configuration.^47^ To establish the catalytic activity of Cj1432, the gene for the N-terminal domain (residues 1–356) was chemically synthesized and expressed in *E. coli*, and the enzyme was purified to homogeneity.

### Catalytic Activity of Cj1432_N_

**Figure 6:**
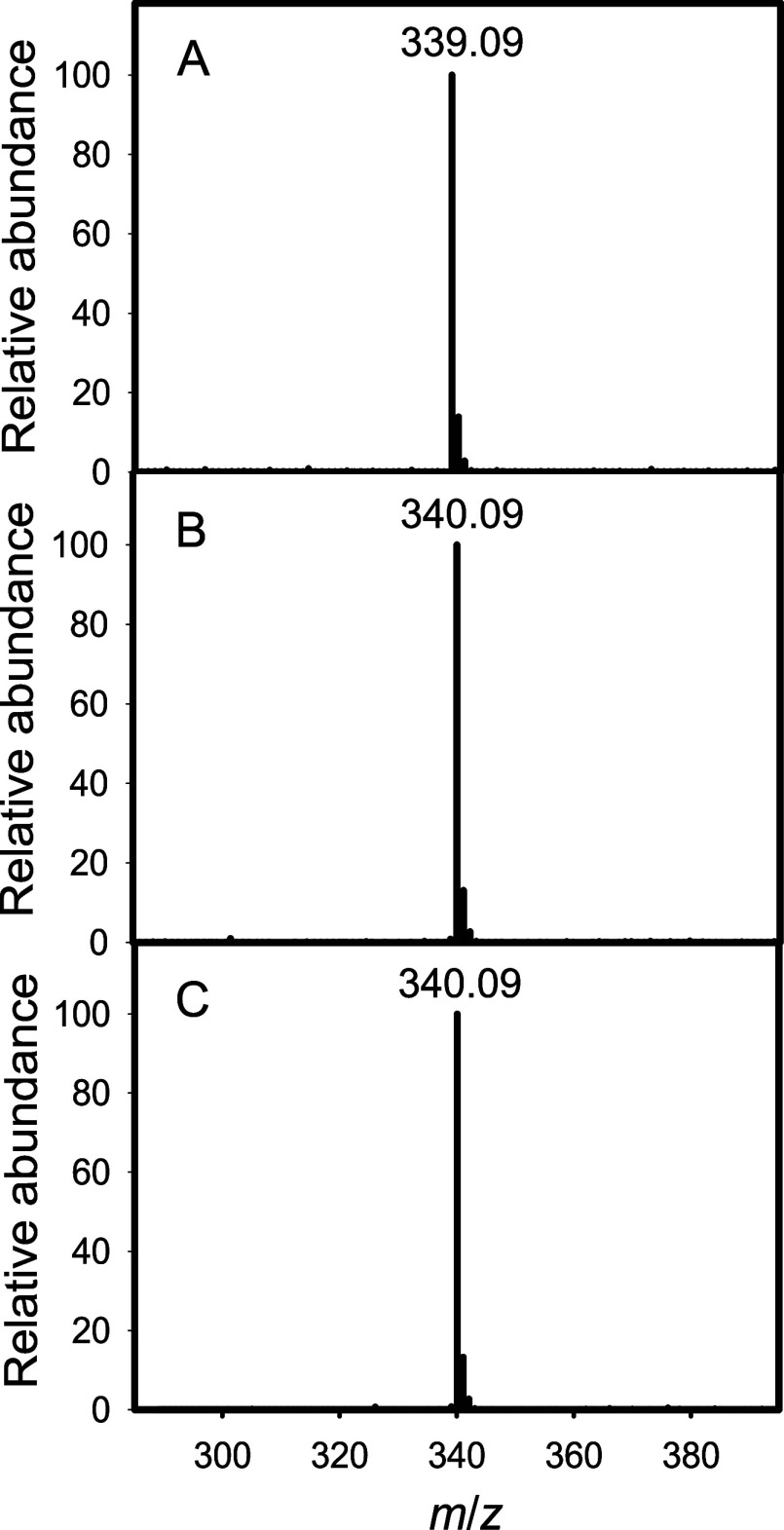
ESI-MS of the Cj1432_N_-catalyzed reaction products. (A) The reaction product (**7a**) formed from UDP-d-GlcA (**2**) and methyl β-d-riboside (**6a**) catalyzed by Cj1432_N_. (B) The reaction product (**7b**) formed from UDP-d-GlcA and methyl [2-^13^C]-β-d-riboside (**6b**) catalyzed by Cj1432_N_. (C) The reaction product (**7c**) formed from UDP-d-GlcA and methyl [3-^13^C]-β-d-riboside (**6c**) catalyzed by Cj1432_N_.

The truncated Cj1432_N_ protein was incubated with UDP-d-GlcA (**2**) and a variety of potential acceptor substrates including d-ribose-5-P, methyl β-d-riboside (**6a**), methyl 2-deoxy-β-d-riboside, and methyl β-d-riboside-5-P in 50 mM NH_4_HCO_3_, pH 8.0, for up to 18 h. These compounds were chosen as the simplest acceptors that were likely to be substrates for the reaction catalyzed by Cj1432_N_. The reaction mixture was initially analyzed using mass spectrometry to detect possible disaccharide formation. Cj1432_N_ was able to catalyze a reaction between UDP-d-GlcA (**2**) and methyl β-d-riboside (**6a**), but with none of the other compounds that were tested. The product was purified using anion exchange chromatography, and the negative ion ESI mass spectrum of the isolated compound is presented in Figure 6A with an *m*/*z* for the M – H anion of 339.09, which is fully consistent with the formation of disaccharide **7a**. The chemical structures of the substrates and products are shown in Figure 3.

**Figure 7:**
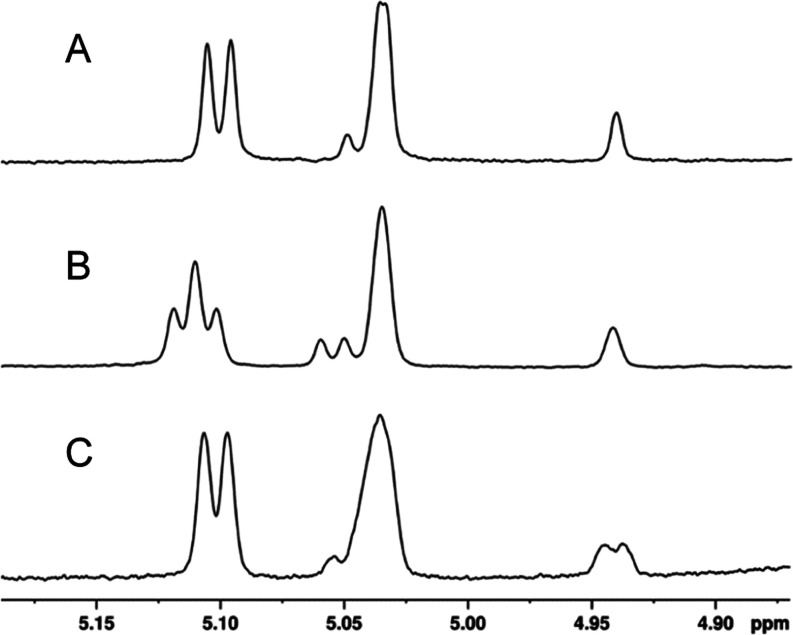
Portion of the ^1^H NMR spectrum of the Cj1432_N_-catalyzed reaction products formed from UDP-d-GlcA (**2**) and methyl β-d-riboside (**6a**, **6b**, and **6c**) highlighting the two anomeric hydrogens of the product disaccharide. (A) Products **7a** and **8a**; (B) products **7b** and **8b**; (C) products **7c** and **8c**. Additional details are provided in the text.

To further confirm the proposed structure of the Cj1432_N_-catalyzed reaction product, the sample was lyophilized, dissolved in D_2_O, and further analyzed using ^1^H NMR spectroscopy. Figure 7A shows a portion of the ^1^H NMR spectrum between 4.90 and 5.20 ppm of the Cj1432_N_ reaction product that highlights the region for the two anomeric hydrogens at C1 for the d-Rib and d-GlcA moieties of the disaccharide product. The full ^1^H NMR spectrum and the HSQC spectrum are found in Figures S4 and S5, respectively. The doublet at ∼5.10 ppm (*J*_1,2_ = 3.8 Hz) and the broad singlet at ∼5.03 ppm (*J*_1,2_ < 1 Hz) are assigned to the anomeric hydrogens of the d-GlcA and d-Rib moieties, respectively, of compound **7a**. The coupling constant of 3.8 Hz for the anomeric hydrogen of the d-GlcA moiety is consistent with an assignment of an α-configuration for disaccharide **7a**. This indicates that the N-terminal domain of Cj1432 catalyzes the transfer of d-glucuronic acid from UDP-α-d-GlcA (**2**) to C2 of a d-ribofuranoside acceptor with retention of configuration.

Two additional minor resonances at ∼5.04 and ∼4.94 ppm are also observed and initially assigned to the anomeric hydrogens of the d-GlcA and d-Rib moieties for an alternate product **8a**, in which the glycosidic bond is formed with the hydroxyl group at C3 of the d-Rib moiety, instead of C2. This situation is perhaps not totally unexpected given the minimal acceptor substrate that was provided for the glycosyltransferase enzyme with the donor UDP-d-GlcA. The fraction of the minor product is ∼18%.

The chemical structures of the reaction products formed from the incubation of UDP-d-GlcA (**2**) and the d-ribose acceptor **6a** were further interrogated using [2-^13^C]-and [3-^13^C]-labeled d-ribose derivatives **6b** and **6c**. The ^1^H NMR spectra of compounds **6b** and **6c** (a mixture of the α- and β-anomers) are presented in Figure S1. When either of these two compounds was used as a substrate with UDP-d-GlcA, the isolated disaccharide was found to have a *m*/*z* of 340.09 for the M-H anion when analyzed by ESI-MS (Figure 6B,C). This is precisely what is expected for the ^13^C-labeled products (**7b**, **7c**, **8b**, and **8c**).

The disaccharide products from the reactions using the d-Rib acceptors **6b** and **6c** with UDP-d-GlcA were also assessed using ^1^H NMR spectroscopy (Figures 7B,C, S6, and S7). The anomeric hydrogen for the d-GlcA moiety of the disaccharide **7b** formed from **6b** at ∼5.11 ppm is now a triplet (coupling constant 3.5 Hz) because of the three-bond coupling with the ^13^C-label at C2 of the d-Rib moiety. The anomeric hydrogen for minor product **8b** remains a doublet at ∼5.05 ppm. Conversely, using the C3-labeled d-Rib acceptor (**6c**), the major disaccharide product **7c** exhibits a doublet for the anomeric hydrogen of the d-GlcA moiety. Unfortunately, the anomeric hydrogen for the d-GlcA moiety for minor product **8c** is obscured by the resonance for the major product of the d-Rib moiety. However, in the NMR spectrum of product **9c**/**10c**, this resonance is clearly observed as a triplet from the 3-bond coupling to the ^13^C-label at C3 (vide infra).

### Determination of the Rate of Reaction Catalyzed by Cj1432_N_

The steady-state rates were determined by measuring the change in the concentration of UDP as a function of time. The concentrations of UDP-d-GlcA and UDP were determined spectrophotometrically at 255 nm using ion exchange chromatography to separate the substrate and product from one another. At a fixed concentration of 2.0 mM UDP-d-GlcA (**2**) and variable concentrations of methyl β-d-riboside (**6a**), the values of *k*_cat_ and *K*_m_ were determined to be 0.51 ± 0.01 min^–1^ and 0.97 ± 0.09 mM, respectively, from fit of the data to the Michaelis–Menten equation (Figure S8).

### Catalytic Activity of Cj1438_C_

The catalytic properties of purified Cj1438_C_ have been reported previously using the methyl glycoside derivative of d-GlcA as the substrate for amide bond formation with ATP and (*S*)-serinol-P or ethanolamine-P.^26^ It was previously shown that UDP-d-GlcA (**2**) is not a substrate for this enzyme and therefore amide bond formation within the d-GlcA moiety of the CPS must occur after the transfer of d-GlcA to the growing polysaccharide chain and not before.^26^ Here, we initially used the product of the condensation reaction between d-GlcA and d-Rib catalyzed by Cj1432_N_ (compound **7a**) as the substrate for amide bond formation catalyzed by Cj1438_C_. This experiment is important because it helps to confirm our previous results, and it enables the chemoenzymatic synthesis of modified disaccharides (for example, **9a** and **11a**) that can be used as potential substrates for additional glycosyltransferases.

**Figure 8:**
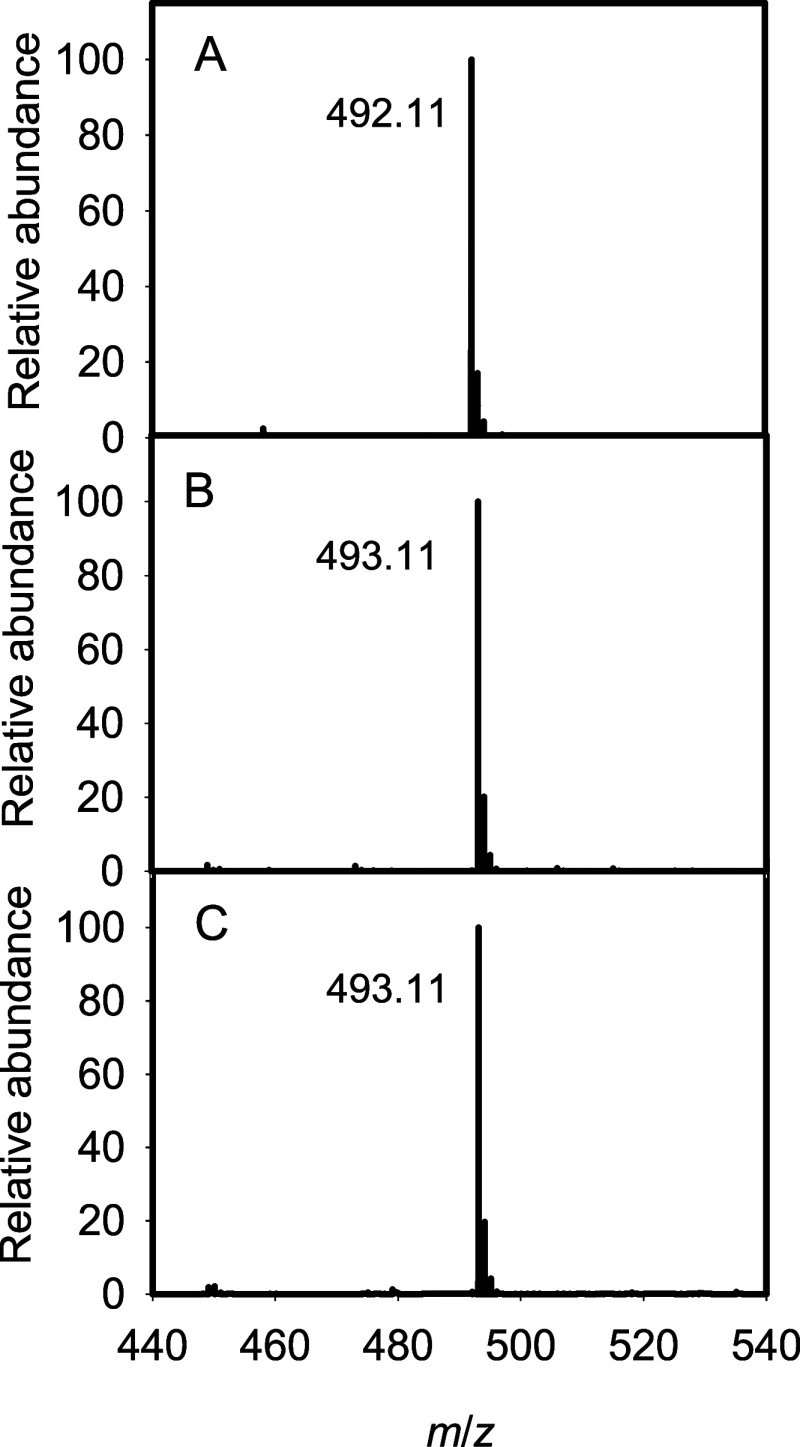
Negative ion ESI-MS of Cj1438_C_-catalyzed reaction products. (A) Cj1438_C_ reaction product (**9a**) using **7a** as the substrate; (B) Cj1438_C_-catalyzed reaction product (**9b**) using **7b** as the substrate; (C) Cj1438_C_-catalyzed reaction product (**9c**) using **7c** as the substrate.

Incubation of Cj1438_C_ with ATP, MgCl_2_, compound **7a**, and (*S*)-serinol-P resulted in the formation of a new compound in addition to the production of ADP. The reaction products were interrogated by negative ion ESI-MS and a new peak was observed at an *m*/*z* of 492.11, which is fully consistent with the formation of compound **9a** (Figure 8A). The Cj1438_C_-catalyzed reaction product was further purified using a HiTrap Q HP anion exchange column. The same reaction was also conducted using ^13^C-labeled compounds **7b** and **7c**. With **7b** or **7c** as the substrate, the resulting products (**9b** or **9c**) were both found to have an *m*/*z* for the M – H anion of 493.11, which is fully consistent with amide bond formation (Figure 8B,C).

**Figure 9:**
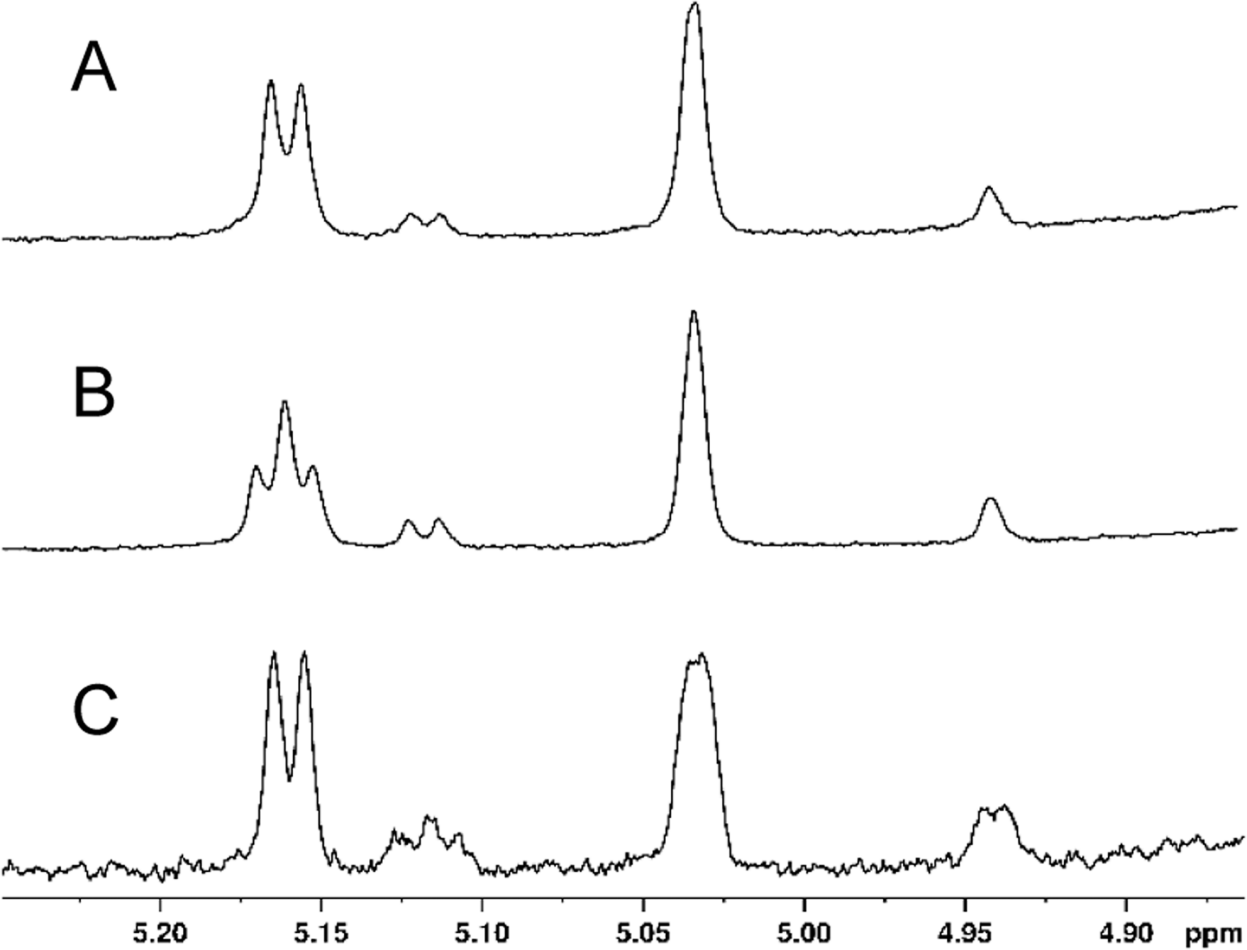
Portion of the ^1^H NMR spectrum of the Cj1438_C_ catalyzed reaction products formed from **7abc**, ATP, and (*S*)-serinol-P. (A) Products **9a** and **10a**; (B) products **9b** and **10b**; (C) products **9c** and **10c**. Additional details are provided in the text.

The products of the reaction catalyzed by Cj1438_C_ using (*S*)-serinol-P, ATP, and either **7a**, **7b**, or **7c** as the carboxylate substrate were isolated and further analyzed by ^1^H- and ^31^P NMR spectroscopy. The selected regions of the ^1^H NMR spectra for the two anomeric hydrogens of the d-GlcA and d-Rib moieties are highlighted in Figure 9 (see also Figures S9–S13 for additional ^1^H NMR, ^31^P NMR, and HSQC spectra of these compounds). These spectra clearly indicate that both the major (C2-riboside, **7a**, **7b**, and **7c**) and minor (C3-riboside, **8a, 8b**, and **8c**) products from the reaction catalyzed by Cj1432_N_ are substrates for the reaction catalyzed by Cj1438_C_. It is also apparent that the resonances for the anomeric hydrogen of the minor d-GlcA moiety are more clearly visible. This change in chemical shift enables the triplet of the minor product (compound **10c**) to be clearly identified (Figure 9C) at ∼5.12 ppm, thus further confirming that the minor product made in the reaction catalyzed by Cj1432_N_ is formed via the attack of the hydroxyl group at C3 of substrate **6** with UDP-d-GlcA.

### Catalytic Activity of Cj1435

The Cj1435 enzyme has been characterized as a member of the HAD phosphatase superfamily and previously shown by us to catalyze the hydrolysis of the phosphate esters from the phosphorylated amides of d-GlcA.^25^ The large HAD superfamily includes phosphatases, phosphoglucomutases, and various dehalogenases.^48^ Here, we used the products of the reactions catalyzed by Cj1438_C_ (compounds **9a**, **9b**, and **9c**) as potential substrates for Cj1435. The reactions contained Cj1435 in the presence of **9a**, **9b**, or **9c** and were allowed to react for 4 h before the reaction products were analyzed by positive ion ESI-MS. Using **9a** as the substrate, a peak at an *m*/*z* of 414.16 was found in the mass spectrum, which is fully consistent with the hydrolysis of **9a** to **11a**. Hydrolysis of either **9b** or **9c** resulted in the formation of a new product with an *m*/*z* of 415.16, fully consistent with the formation of **11b** or **11c**. The ^1^H NMR and HSQC spectra are shown in Figures S14 and S15 for product **11a**.

### Biosynthetic Pathway for CPS Polymerization

**Figure 10:**
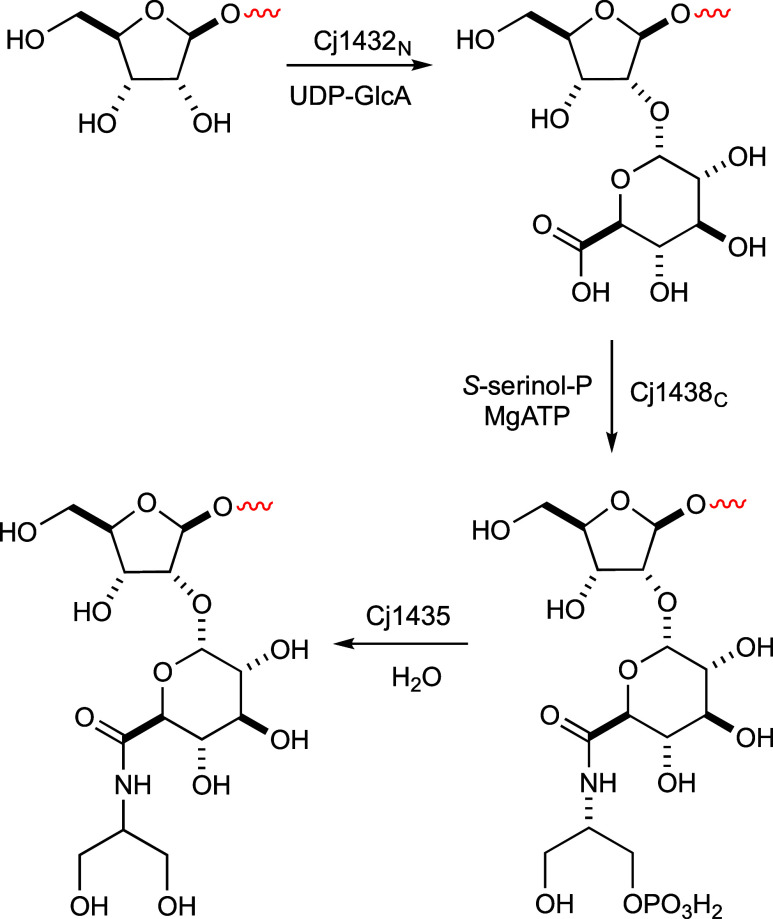
Reactions catalyzed by Cj1432_N_, Cj1438_C_, and Cj1435 during the biosynthesis of the capsular polysaccharide of *C. jejuni* NCTC 11168.

We have identified for the first time a glycosyltransferase that is required for polymerization of the CPS in the human pathogen *C. jejuni*. The N-terminal domain of Cj1432 is shown here to catalyze the formation of the glycosidic bond between d-glucuronate and the C2 hydroxyl group of d-ribose. The product of this reaction is the substrate for the enzyme that comprises the C-terminal domain of Cj1438, which catalyzes the ATP-dependent formation of an amide bond of (*S*)-serinol-P with the C6 carboxylate of d-GlcA. The phosphorylated product of this reaction is subsequently hydrolyzed by Cj1435. The biosynthetic steps are summarized in Figure 10.

## Conclusions

The exterior surface of human pathogen *C. jejuni* is encased by a capsular polysaccharide that helps protect the bacterium from the host immune system. The CPS is composed of a repeating unit of 2–5 different monosaccharides that can be decorated by various modifications to the carbohydrate structure. Much information has been gathered previously regarding the biosynthesis of individual monosaccharides and subsequent modifications, but the enzymes required for the polymerization of the CPS have remained elusive. Here, we have demonstrated that the N-terminal domain of Cj1432 from *C. jejuni* NCTC 11168 (serotype HS:2) catalyzes the condensation of the C2 hydroxyl group of d-ribofuranose with UDP-d-GlcA. This represents the first characterization of a glycosyltransferase from *C. jejuni* that is required for the polymerization of the capsular polysaccharide. It was further demonstrated that the product of the reaction catalyzed by Cj1432 is the substrate for Cj1438, which catalyzes amide bond formation using the C6 carboxylate of the GlcA moiety, (*S*)-serinol-P and ATP. The product of the reaction catalyzed by Cj1438 is the substrate for Cj1435, which catalyzes the hydrolysis of phosphate from the serinol group. These results further demonstrate that the amide bond decoration occurs after incorporation into the growing polysaccharide chain and may be required prior to the next polymerization step.

## Supporting Information Available

Amino acid sequences of the purified proteins and NMR spectra of chemically synthesized substrates and enzyme-catalyzed reaction products (PDF)
