# Supplementary material for: Identification of the Polymerizing Glycosyltransferase Required for the Addition of d-Glucuronic Acid to the Capsular Polysaccharide of Campylobacter jejuni
Source: Biochemistry. 2025 Jan 24;64(3):581–90. doi: 10.1021/acs.biochem.4c00703 (PMC11800397; doi:10.1021/acs.biochem.4c00703)
Supplement: Supplementary file 1 — bi4c00703_si_001.pdf [file bi4c00703_si_001.pdf]

SUPPLEMENTARY INFORMATION

Identification of the Polymerizing Glycosyltransferase Required for the Addition of D-Glucuronic Acid to the Capsular Polysaccharide of *Campylobacter jejuni*

Dao Feng Xiang[ϕ], Alexander S. Riegert[§], Tamari Narindoshvili[ϕ] ,
and
Frank M. Raushel[ϕ,*]

[§]Department of Biochemistry & Biophysics, Texas A&M University, College Station, TX 77843, United States.

[ϕ]Department of Chemistry, Texas A&M University, College Station, TX, 77843, United States.

*Contact Information
e-mail: raushel@tamu.edu
phone: 1-979-845-3373

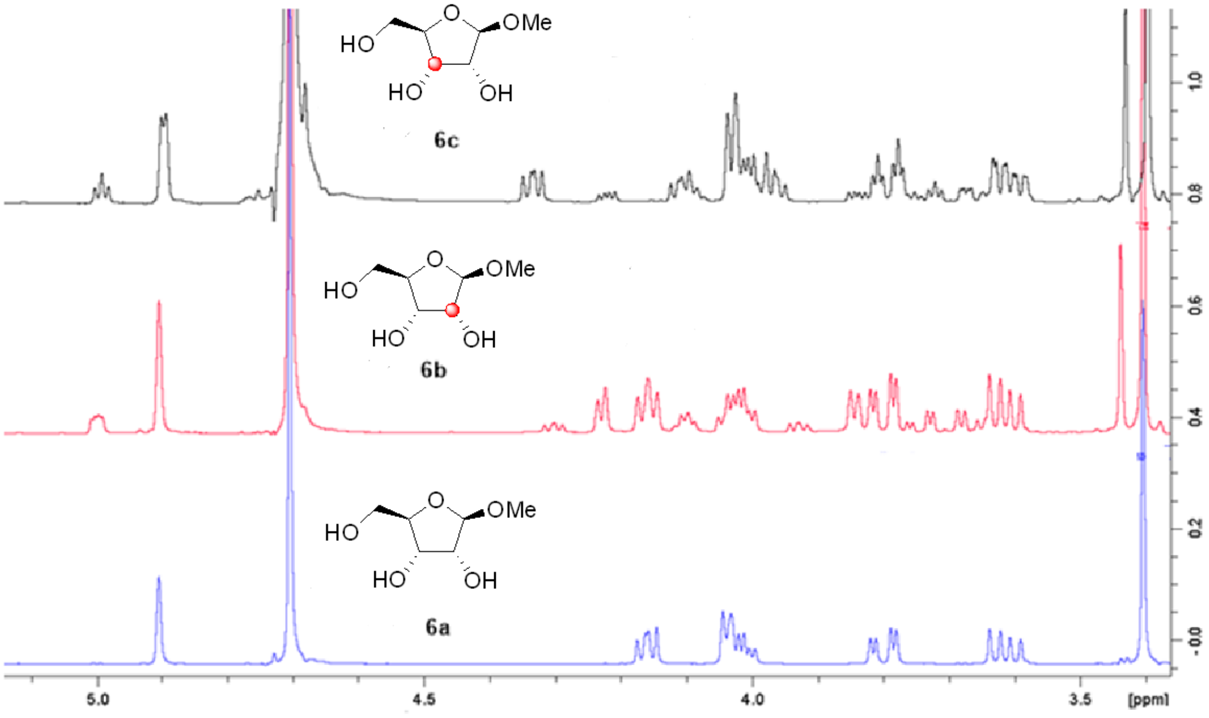

Figure S1: ^{1}H NMR spectra of compounds **6a** (blue), **6b** (red), and **6c** (black). The red dot indicates the position of a ^{13}C label. The methyl-β-D-riboside (**6a**) was purchased as the single β-anomeric form, whereas **6b** and **6c** were isolated with ~20% of the α-anomer.

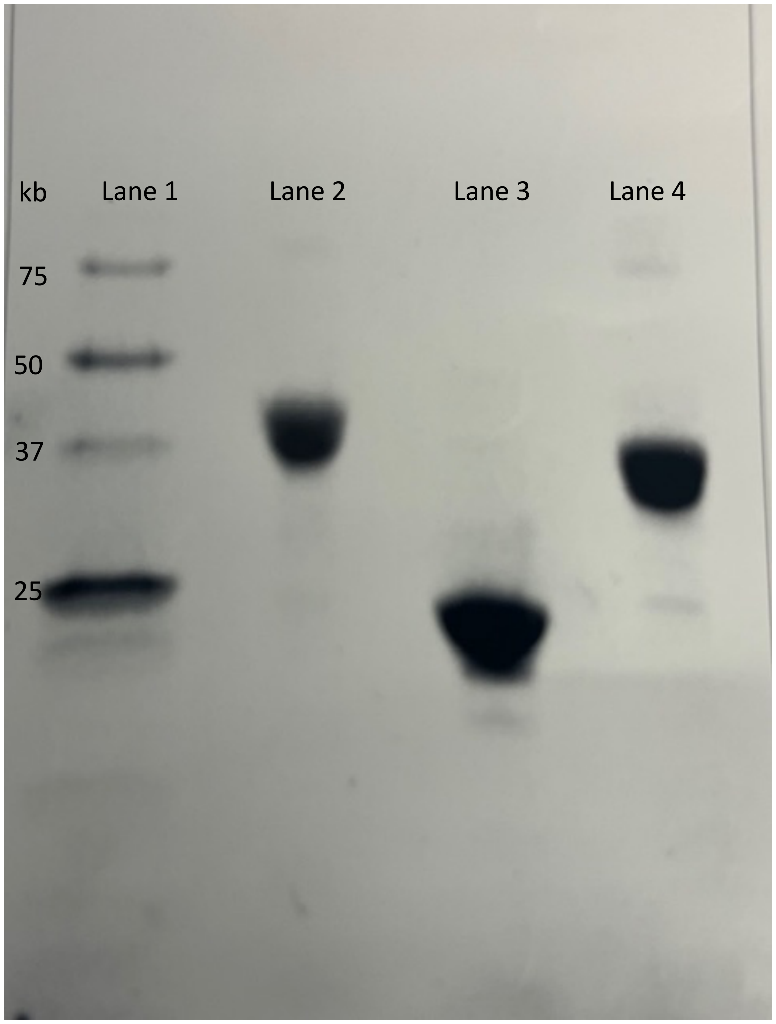

Figure S2. SDS gel of the enzymes purified for this investigation. Lane 1: Precision Plus Protein™ Standards. Lane 2: $Cj1432_N$, Lane 3: Cj1435, and Lane 4: $Cj1438_C$.

(a)

MGSSHHHHHHSSENLYFQGHMIKKILLITPELEYTGALNSFKRICEVLLNNKYAVDIWTYNEGP
YISEFDKLGVYVEVISEDDIDSKWVHERISKYSLVIANTIVVYKCVELIQNLTPVVWYIREAEN
LPDFFWKPERKLALEKAKKLYVVSEYAKDFIIHNYNKNVEVLHNYVDDVFYEKHDDFLKQIKSD
KLKFLALGTIEKRKGYDVLLQAFIDLPVDIRDQCELHFAGRFWEGAKDFFPKILSLAKKFPNIF
YHGELRDRKKIHSLIFQCNVMVVPSRDESCSLVALEGAMMSKPLILTENIGAKYILDENSGWLV
KTGSVDSLKNAFIQAYKNKNKLDAMGANSRNNYLQTSTYEIYEKNILKMVRDEICK

(b)

MKELSDYDFLLNRHKQIFDYTPDFKCPVTFNEKLIYRILYDRSCIYSFLADKIKMRFYVASALS
DNHEYSWDKIDILNEKSILFNNIDDLQDKIFETNKCKYLPKIYGIYKNIYDINFNELPNSFVLK
TNHDCGGYVIVENKQEFLRDTVVFSNAMKKLKKHLEWNYYSVFREWHYKDIEPRVFAEELLLGE
NKKPADTYKFHIFDKENLSNNFIQVTTDRFDNYQRAMFDLSWNLAPFNFMYDNKNVTMIPKKPN
LLDSMINISLILAKPFDYVRVDLYQFDKKIYIGELTFTHGAAGEKVIPKEWDKKLGDLWRLKRL
DNASKLEHHHHHH

(c)

MSKEILALFDFCETLTNFQTLDRYLPLAGSKNINYTQSKNLARRERFQRENLPYPRYEWLIDLD
VDLAEEIAQEFVYTDVMANLNQNVMDRLFWHQDEGHTIVIVSGGLTIYIKEFARIYNIENIVAV
DLEIYKNKLTGNIDGIHTMQERKLYKLAQKFNLKQFDLKNSYAYSDCVSDIPLLSLVGNPNVIE
CGKDLQWARILGFNILLKYLEHHHHHH

Figure S3. Amino acid sequences of the proteins used for this investigation. (a) Amino acid sequence for $Cj1432_N$. The polyhistidine tag at the beginning is shown in red font. (b) Amino acid sequence for $Cj1438_C$. The added polyhistidine purification tag at the C-terminal end is shown in red font. (c) The amino acid sequence for Cj1435. The added polyhistidine purification tag at the C-terminal end is shown in red font.

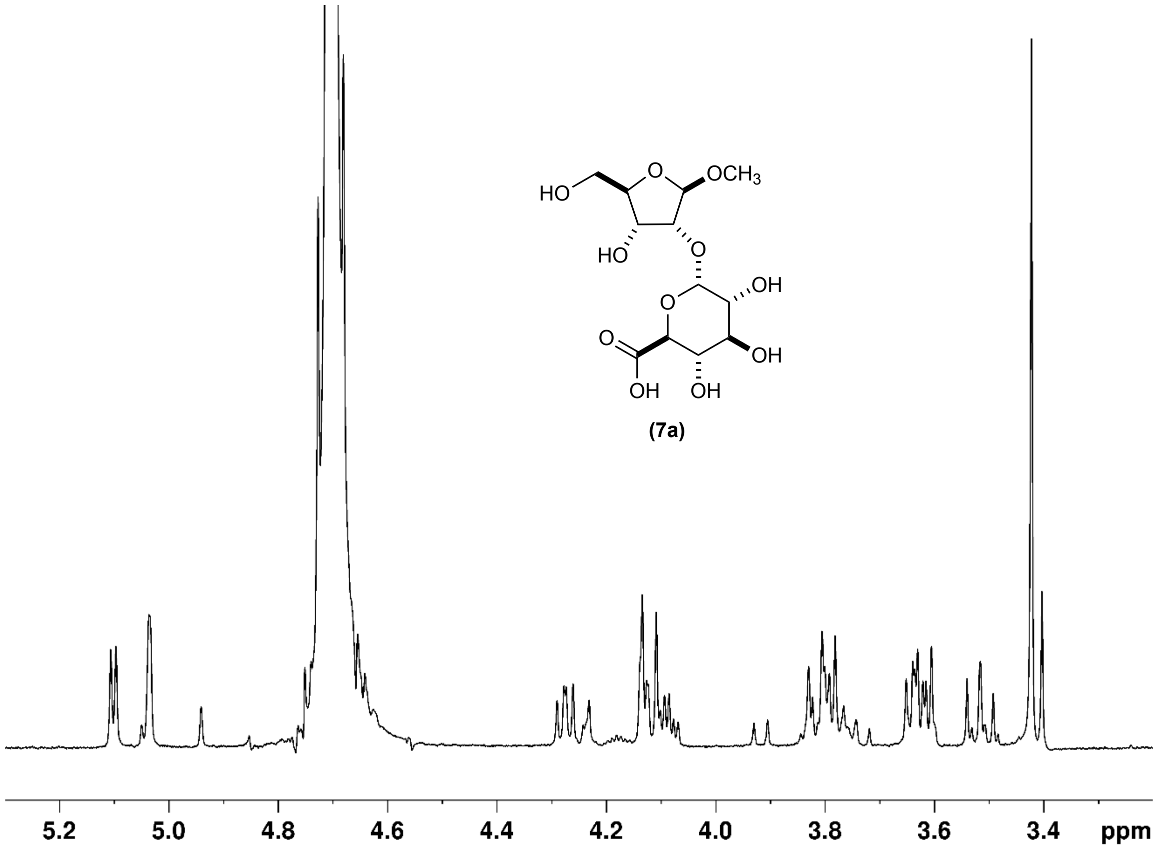

Figure S4. ^{1}H NMR spectrum of product **7a** in D_2O from the reaction catalyzed by Cj1432$_N$ using substrates UDP-GlcA (**2**) and compound **6a**. The minor product (~18%) is compound **8a**.

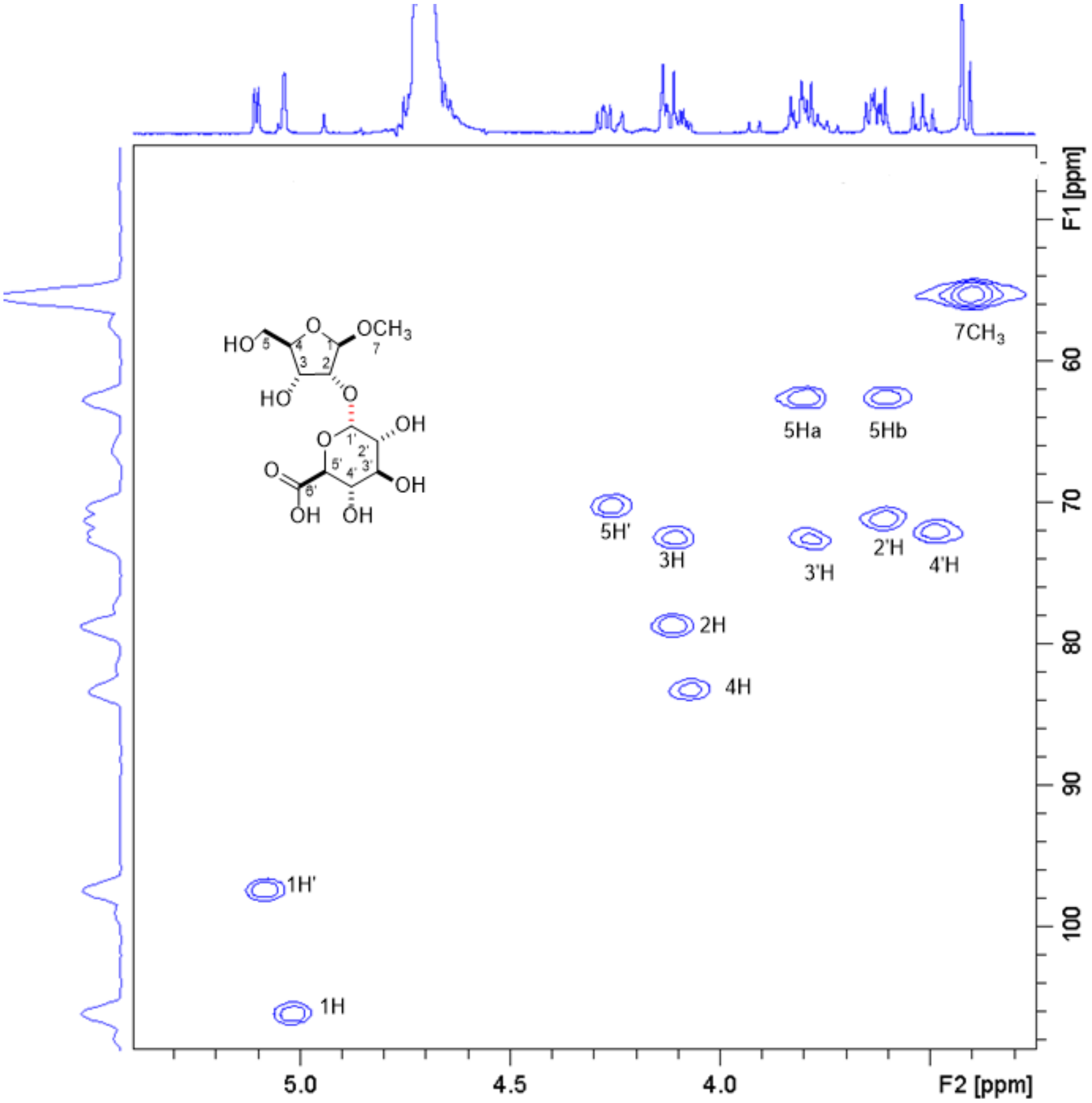

Figure S5: HSQC spectrum of product **7a** in D_2O from the reaction catalyzed by $Cj1432_N$ using substrates UDP-GlcA (**2**) and compound **6a**.

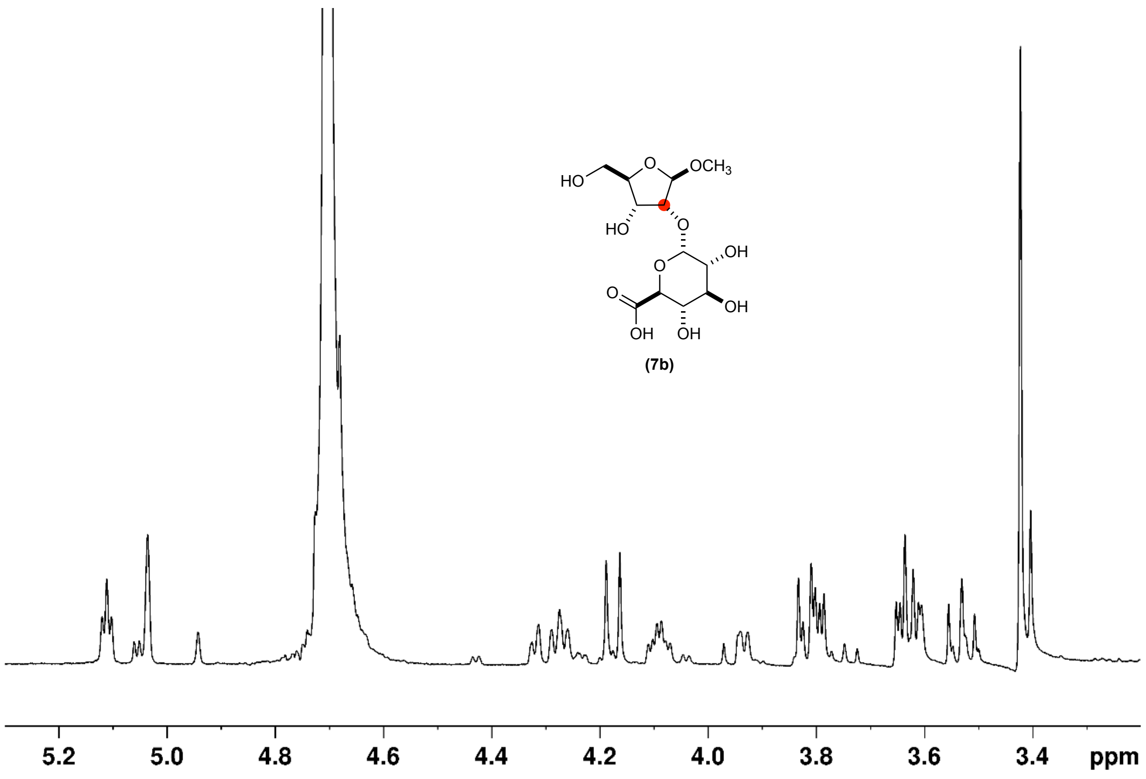

Figure S6: ^{1}H NMR spectrum of product **7b** in D_2O from the reaction catalyzed by Cj1432$_N$ using substrates UDP-GlcA (**2**) and compound **6b.** The red dot indicates the site of a ^{13}C-label. The minor product (~17%) is compound **8b**.

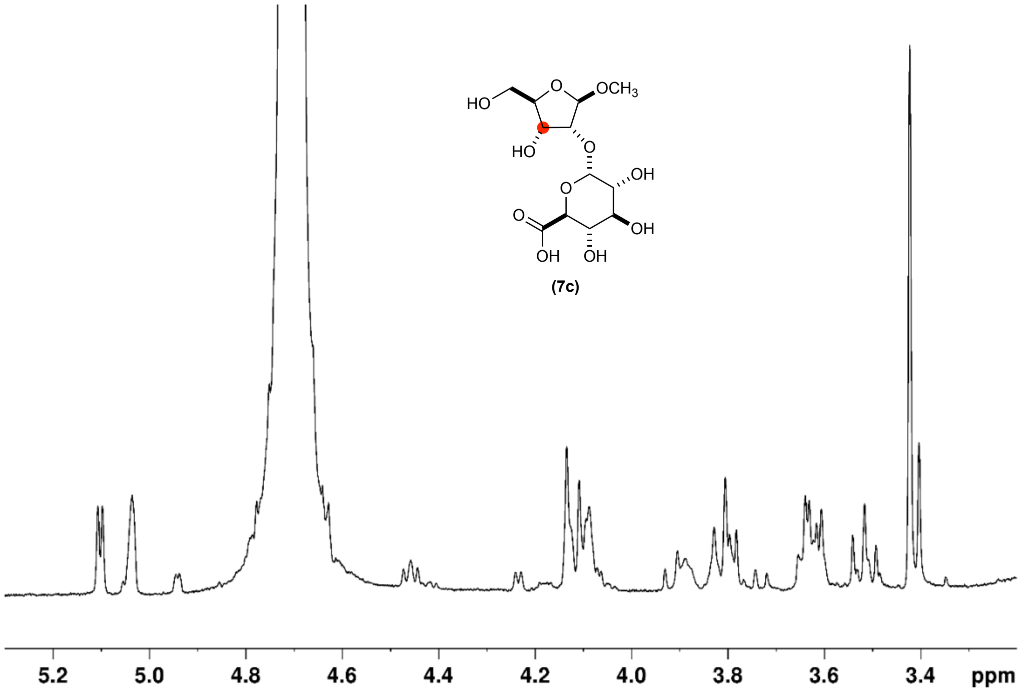

Figure S7: ^{1}H NMR spectrum of product **7c** in D_2O from the reaction catalyzed by Cj1432$_N$ using substrates UDP-GlcA (**2**) and compound **6c**. The red dot indicates the site of a ^{13}C-label. The minor product (~21%) is compound **8c**.

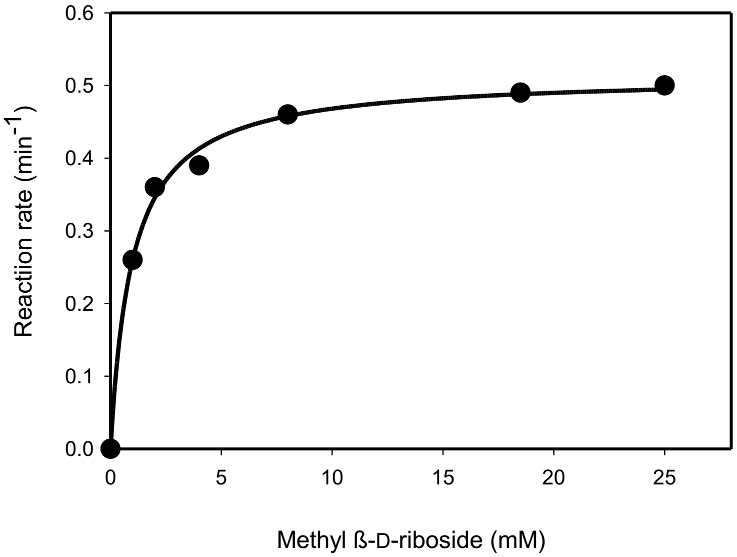

Figure S8: Steady state kinetic analysis of the reaction catalyzed by Cj1432$_N$. Reaction conditions: Cj1432$_N$ (10 μM), 2.0 mM UDP-GlcA, 50 mM NH_4HCO_3, pH 8.0, and variable concentrations of methyl β-D-riboside (0-25 mM). The rate of the reaction was monitored by following the formation of UDP at 255 nm via anion exchange chromatography as a function of time. The data were fit to the equation: $v/E_t = k_{cat}(S) / (K_m + S)$ to obtain values for k_{cat} (0.51 ± 0.01 min^{-1}) and K_m (0.97 ± 0.09 mM).

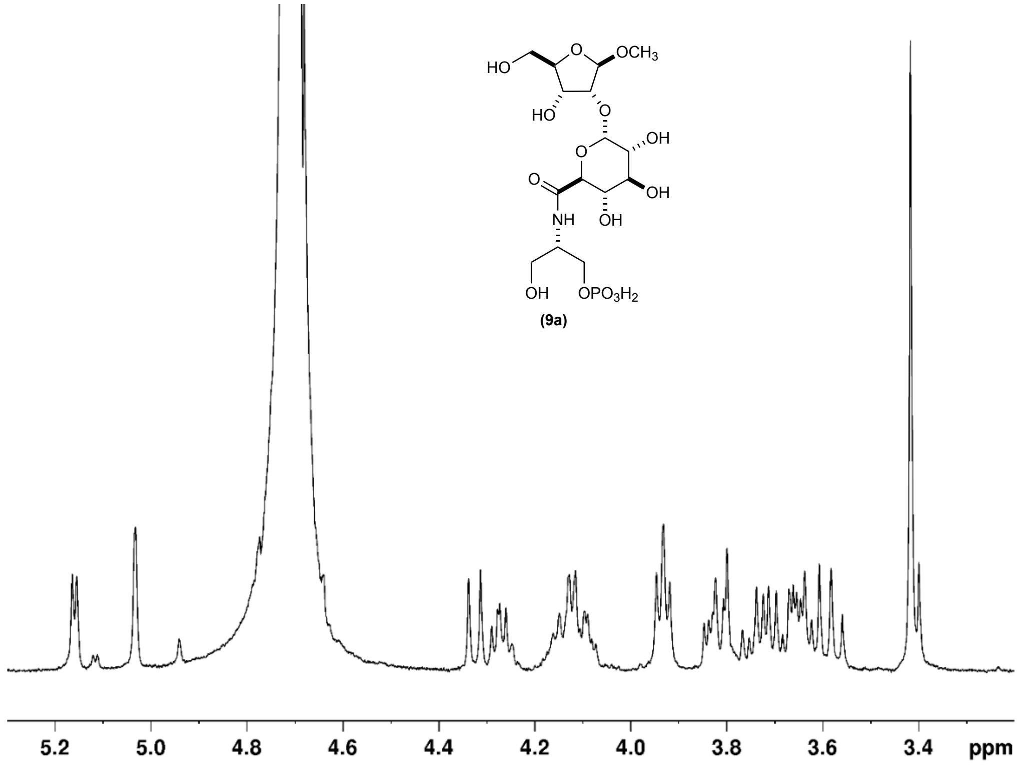

Figure S9: ^{1}H NMR spectrum of product **9a** in D_2O from the reaction catalyzed by Cj1438$_C$ using **7a**, ATP, and *S*-serinol-P as substrates. The minor product (~17%) is compound **10a**.

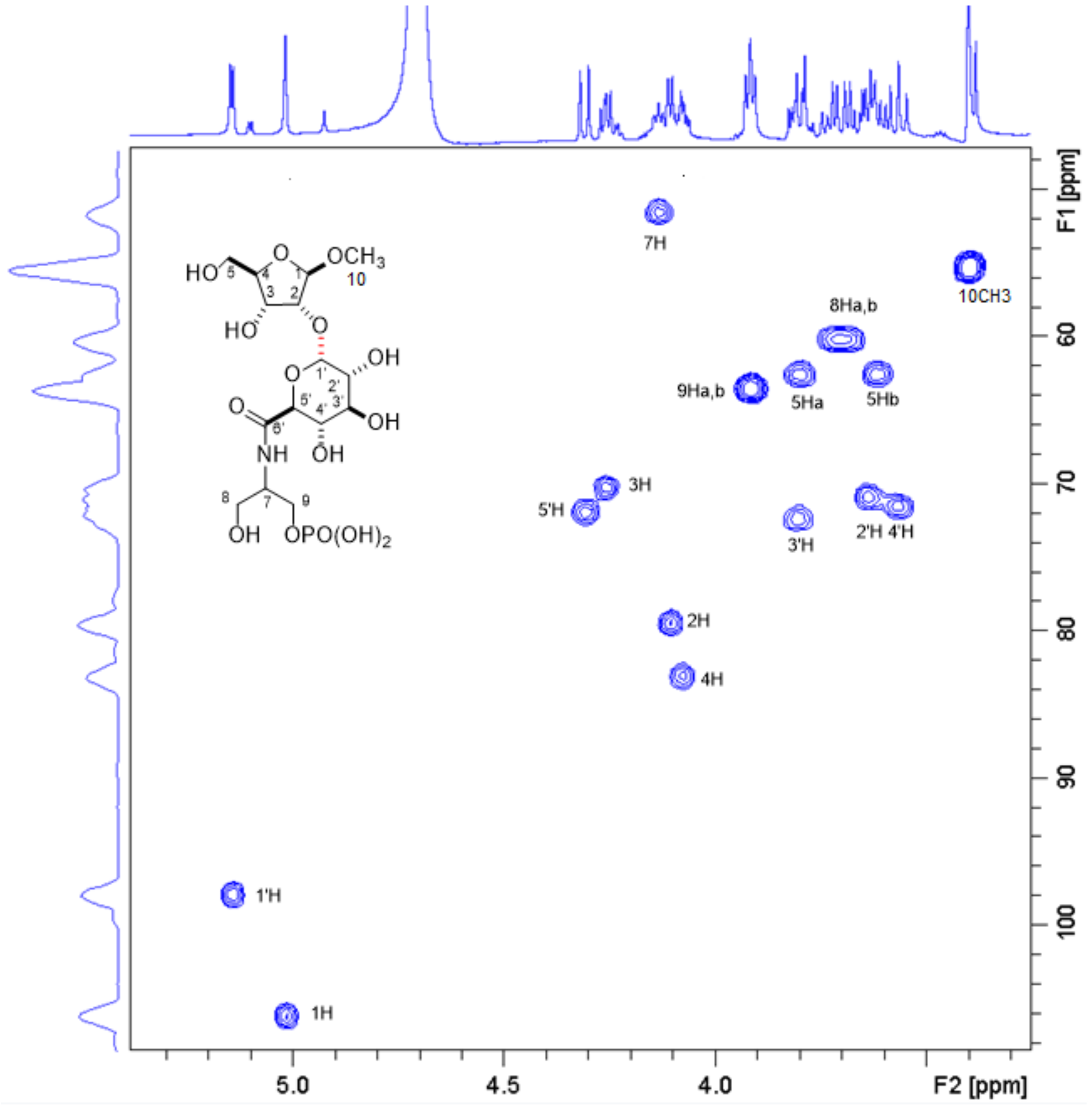

Figure S10: HSQC spectrum of product **9a** in D_2O from the reaction catalyzed by $Cj1438_C$ using **7a**, ATP, and *S*-serinol-P as substrates.

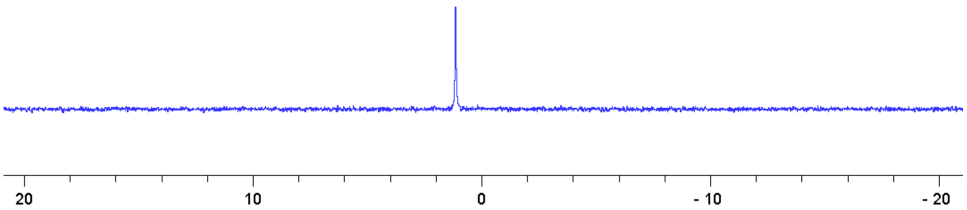

Figure S11. ^{31}P HMR spectrum of product **9a** in D_2O from the reaction catalyzed by Cj1438$_C$ using **7a**, ATP, and *S*-serinol-P as substrates.

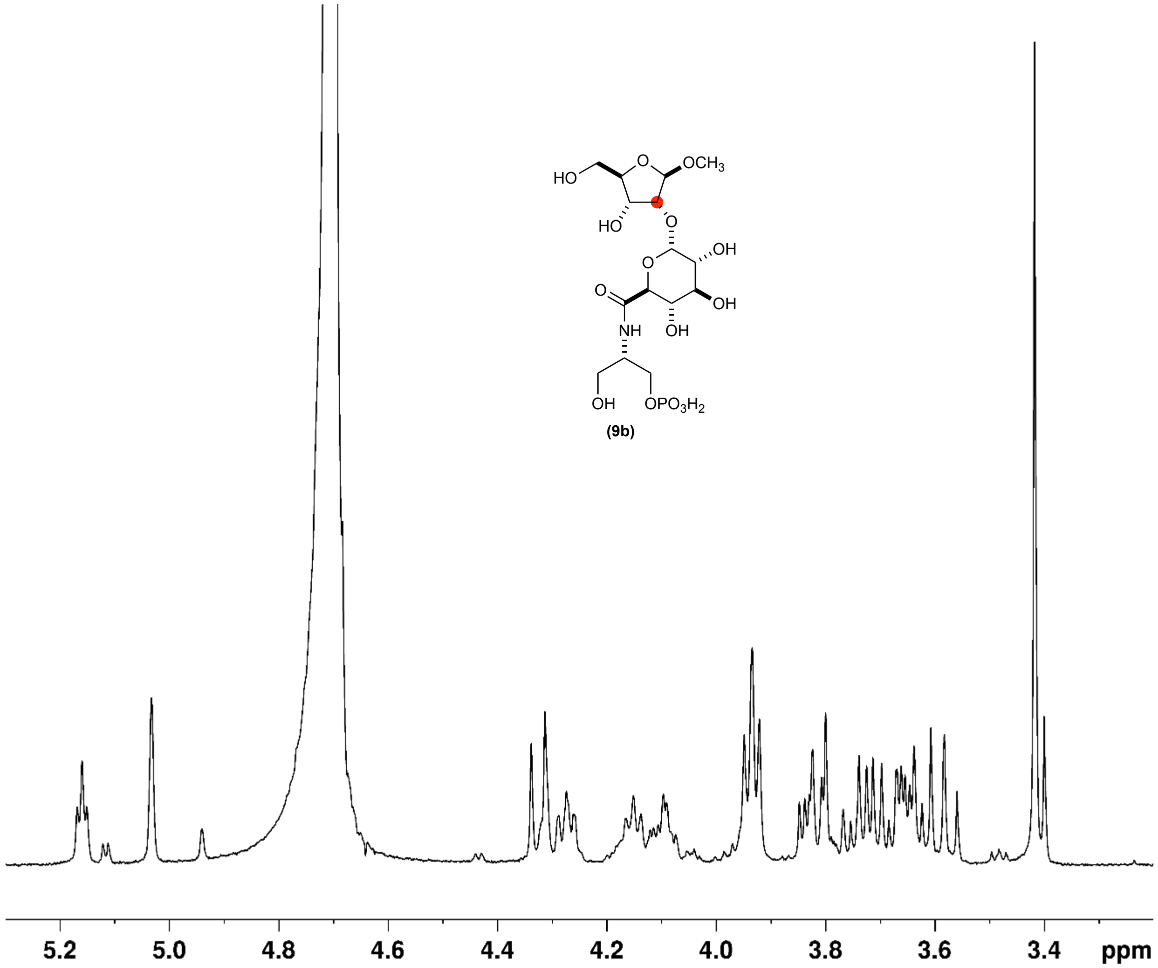

Figure S12. ^{1}H NMR spectrum of product **9b** in D_2O from the reaction catalyzed by Cj1438$_C$ using **7b**, **ATP**, and *S*-serinol-P as substrates. The minor product (~17%) is compound **10b**.

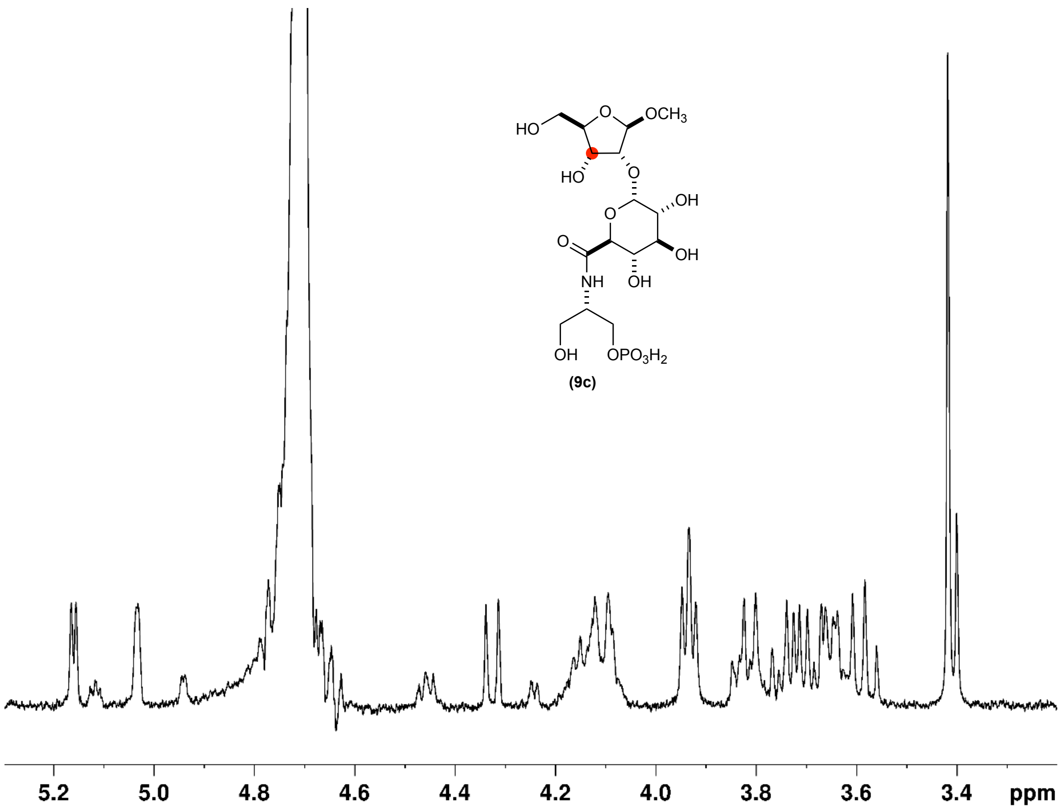

Figure S13: ^{1}H NMR spectrum of product **9c** in D_2O from the reaction catalyzed by Cj1438$_C$ using **7c**, ATP, and *S*-serinol-P as substrates. The minor product (~19%) is compound **10c**.

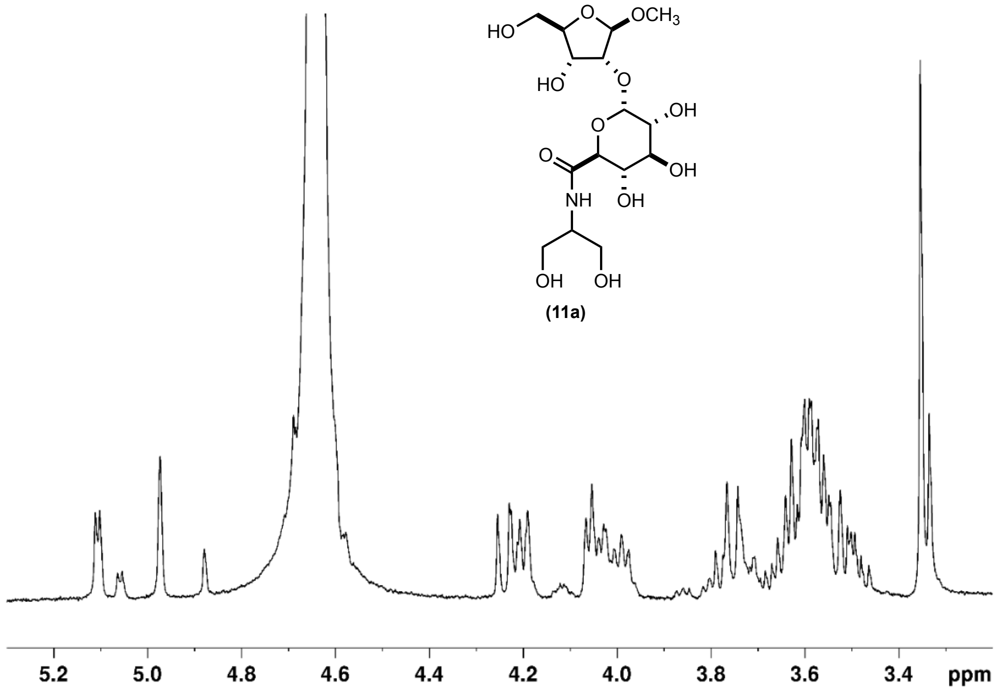

Figure S14: ^{1}H NMR spectrum of product **11a** in D_2O from the reaction catalyzed by Cj1435 using **9a** as the substrate. The minor product (~20%) is compound **12a**.

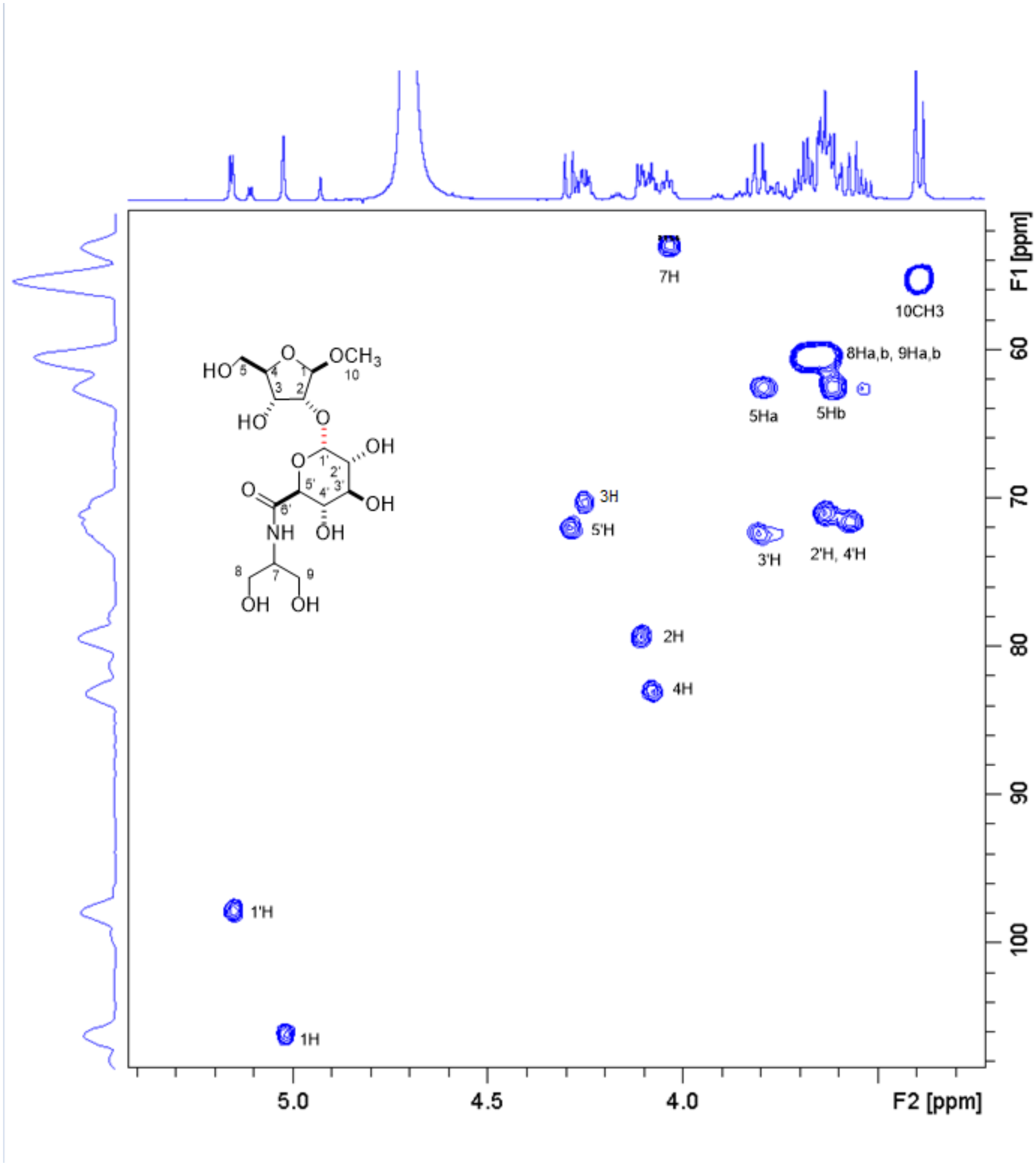

Figure S15: HSQC spectrum of product **11a** in D_2O from the reaction catalyzed by Cj1435 using **9a** as the substrate.
